# mGlu_1_ potentiation enhances prelimbic somatostatin interneuron activity to rescue schizophrenia-like physiological and cognitive deficits

**DOI:** 10.1016/j.celrep.2021.109950

**Published:** 2021-11-02

**Authors:** James Maksymetz, Nellie E. Byun, Deborah J. Luessen, Brianna Li, Robert L. Barry, John C. Gore, Colleen M. Niswender, Craig W. Lindsley, Max E. Joffe, P. Jeffrey Conn

**Affiliations:** 1 Department of Pharmacology, Vanderbilt University, Nashville, TN 37232, USA; 2 Warren Center for Neuroscience Drug Discovery, Vanderbilt University, Nashville, TN 37232, USA; 3 Vanderbilt University Institute of Imaging Science, Vanderbilt University Medical Center, Nashville, TN 37232, USA; 4 Vanderbilt University, Nashville, TN 37232, USA; 5 Department of Radiology & Radiological Sciences, Vanderbilt University Medical Center, Nashville, TN 37232, USA; 6 Department of Biomedical Engineering, Vanderbilt University, Nashville, TN 37232, USA; 7 Vanderbilt Kennedy Center, Vanderbilt University Medical Center, Nashville, TN 37232, USA; 8 Vanderbilt Institute for Chemical Biology, Vanderbilt University, Nashville, TN 37232, USA; 9 Vanderbilt Brain Institute, Vanderbilt University, Nashville, TN 37232, USA; 10 Department of Chemistry, Vanderbilt University, Nashville, TN 37232, USA; 11 Present address: Department of Neuroscience, Genentech, Inc., South San Francisco, CA, USA; 12 Present address: U.S. Department of Health and Human Services, Washington, DC, USA; 13 Present address: Department of Radiology, Massachusetts General Hospital & Harvard Medical School, Boston, MA, USA; 14 Present address: Department of Psychiatry, University of Pittsburgh, Pittsburgh, PA, USA; 15 Lead contact

## Abstract

Evidence for prefrontal cortical (PFC) GABAergic dysfunction is one of the most consistent findings in schizophrenia and may contribute to cognitive deficits. Recent studies suggest that the mGlu_1_ subtype of metabotropic glutamate receptor regulates cortical inhibition; however, understanding the mechanisms through which mGlu_1_ positive allosteric modulators (PAMs) regulate PFC microcircuit function and cognition is essential for advancing these potential therapeutics toward the clinic. We report a series of electrophysiology, optogenetic, pharmacological magnetic resonance imaging, and animal behavior studies demonstrating that activation of mGlu_1_ receptors increases inhibitory transmission in the prelimbic PFC by selective excitation of somatostatin-expressing interneurons (SST-INs). An mGlu_1_ PAM reverses cortical hyperactivity and concomitant cognitive deficits induced by *N*-methyl-d-aspartate (NMDA) receptor antagonists. Using *in vivo* optogenetics, we show that prelimbic SST-INs are necessary for mGlu_1_ PAM efficacy. Collectively, these findings suggest that mGlu_1_ PAMs could reverse cortical GABAergic deficits and exhibit efficacy in treating cognitive dysfunction in schizophrenia.

## In brief

Maksymetz et al. demonstrate that metabotropic glutamate receptor 1 (mGlu-_1_) activation augments somatostatin interneuron (SST-INs) output to increase inhibition in the rodent prelimbic cortex. mGlu_1_ positive allosteric modulators (PAMs) reverse schizophrenia-like cortical hyperactivity and working memory deficits via SST-INs, validating mGlu_1_ as a therapeutic target for cognitive deficits in schizophrenia.

## Graphical Abstract

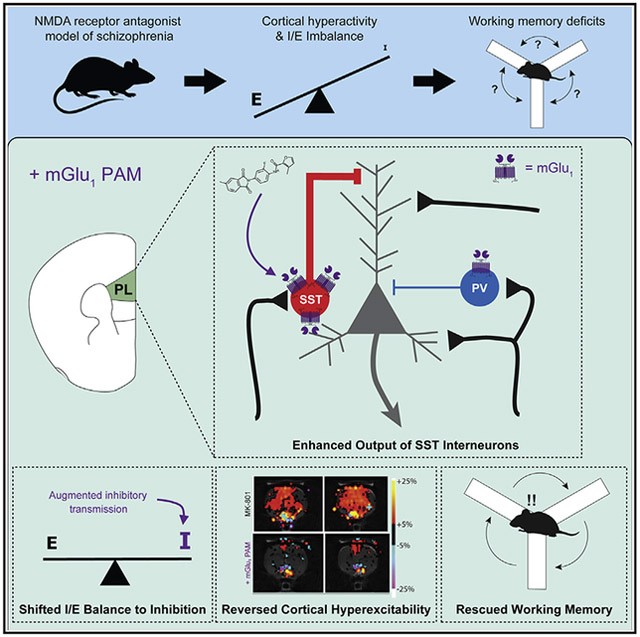

## INTRODUCTION

Available antipsychotic medications reduce the positive symptoms of schizophrenia in some patients but there remains a critical unmet need for therapeutics to treat the negative symptoms and cognitive deficits in this debilitating neuropsychiatric disorder (Bobes et al., 2007; Hill et al., 2010). Chronic cognitive dysfunction strongly predicts long-term functional outcomes for patients with schizophrenia, interfering with employment and interpersonal relationships (Green, 2016). While contemporary antipsychotics minimize or prevent psychosis in many patients by modulating monoaminergic systems, recent evidence suggests that these approaches may exacerbate cognitive impairments (Husa et al., 2017). Therefore, identifying novel ther apeutic targets is necessary to develop more efficacious treatments for all symptom domains of schizophrenia.

Cognitive deficits include impairments in working memory and attention that depend on the function of the dorsolateral prefrontal cortex (dlPFC). Suggestive of cortical dysfunction, one of the most consistent pathophysiological findings in postmortem brain tissue from patients with schizophrenia is a reduction in glutamic acid decarboxylase 67 (GAD67) mRNA and protein in the dlPFC (Lewis et al., 2012). GAD67 produces the inhibitory neurotransmitter γ-aminobutyric acid (GABA), and clinical and preclinical studies support the hypothesis that dysfunction of GABAergic inhibition plays a critical role in the pathophysiology underlying cognitive deficits in schizophrenia (Dienel and Lewis, 2019). Thus, enhancing cortical GABAergic interneuron function represents a promising therapeutic strategy to address unmet clinical needs for people living with schizophrenia.

GABAergic inhibitory interneurons in the medial prefrontal cortex (mPFC) can be subdivided into subclasses based on physiology, morphology, and the expression of molecular markers, with most expressing either somatostatin (SST), parvalbumin (PV), or the serotonin receptor 3a with minimal overlap (Rudy et al., 2011). While fast-spiking PV interneurons (PV-INs) have historically garnered the most attention in schizophrenia research (Gonzalez-Burgos et al., 2015), recent findings suggest a significant role of the lesser studied SST interneurons (SST-INs) in mPFC function, working memory, and the pathophysiology of schizophrenia. Acute inhibition of mPFC SST-INs impairs mPFC-dependent behaviors (Abbas et al., 2018; Cummings and Clem, 2020), suggesting their involvement in higher order cognitive function. Acute administration of an *N*-methyl-d-aspartate (NMDA) receptor antagonist used to model cortical inhibitory deficits (Homayoun and Moghaddam, 2007) also suppresses mPFC SST-IN activity and produces schizophrenia-like behavioral deficits (Ali et al., 2020). Multiple studies have found reduced SST mRNA in postmortem brain tissue from patients with schizophrenia (Fung et al., 2010; Hashimoto et al., 2008), specifically in working memory networks (Tsubomoto et al., 2019), implicating their potential dysfunction in the disease. These findings suggest that enhancing mPFC SST-IN function may be effective in rescuing GABAergic and cognitive deficits in schizophrenia.

Early studies in rodents and non-human primates revealed that the Gα_q/11_-coupled metabotropic glutamate (mGlu) receptor subtype 1 (mGlu_1_) is expressed in mPFC GABAergic interneurons (López-Bendito et al., 2002; Muly et al., 2003; Tasic et al., 2018). While not yet examined in mPFC, mGlu_1_ is highly expressed in SST-INs in the hippocampus and other cortical regions (Baude et al., 1993; Ferraguti et al., 2004). Activation of postsynaptic group I mGlu_1_ receptors, mGlu_1_ and mGlu_5_, can increase neuronal activity (Maksymetz et al., 2017; Mannaioni et al., 2001), raising the possibility that mGlu_1_ activators could enhance cortical SST-IN output. Consistent with this hypothesis, mGlu_1_ receptor activation enhances feedforward inhibition onto glutamatergic pyramidal neurons in acute mPFC slices (Sun and Neugebauer, 2011). The mechanisms through which mGlu_1_ receptors regulate mPFC interneurons are especially interesting in light of human genetic studies revealing multiple loss-of-function mutations in the human mGlu_1_ gene, *GRM1*, associated with schizophrenia (Ayoub et al., 2012; Cho et al., 2014; Frank et al., 2011). This suggests that reduced mGlu_1_ signaling may contribute to disease pathology in some patients. However, the precise role of mGlu_1_ in regulating inhibitory transmission in the mPFC and the potential of selective mGlu_1_ activators to reverse cognitive deficits in schizophrenia models have not been evaluated.

Recently, our group and others have discovered highly selective mGlu_1_ positive allosteric modulators (PAMs) (Cho et al., 2014; Garcia-Barrantes et al., 2015, 2016). We reported that the mGlu_1_ PAM VU6004909 achieves high brain exposure after systemic administration and reverses amphetamine-induced hyperlocomotion and disruption of sensorimotor gating in rodents, suggesting potential antipsychotic-like efficacy (Yohn et al., 2020). If mGlu_1_ PAMs can reverse deficits in mPFC inhibitory transmission, these combined actions could be an alternative approach to reduce positive symptoms and treat cognitive deficits in patients with schizophrenia, providing an exciting improvement over existing antipsychotics. Thus, we took advantage of this mGlu_1_ PAM, along with electrophysiological, pharmacological magnetic resonance imaging, behavioral, and *in vivo* optogenetic approaches, to test this hypothesis. We find that potentiating mGlu_1_ function augments SST-IN output, enhances prelimbic (PL) PFC inhibitory transmission, and reverses deficits in an NMDA receptor hypofunction model of cortical cognitive impairments.

## RESULTS

### mGlu_1_ expression is enriched in PL PFC SST-expressing interneurons

**Figure 1.**
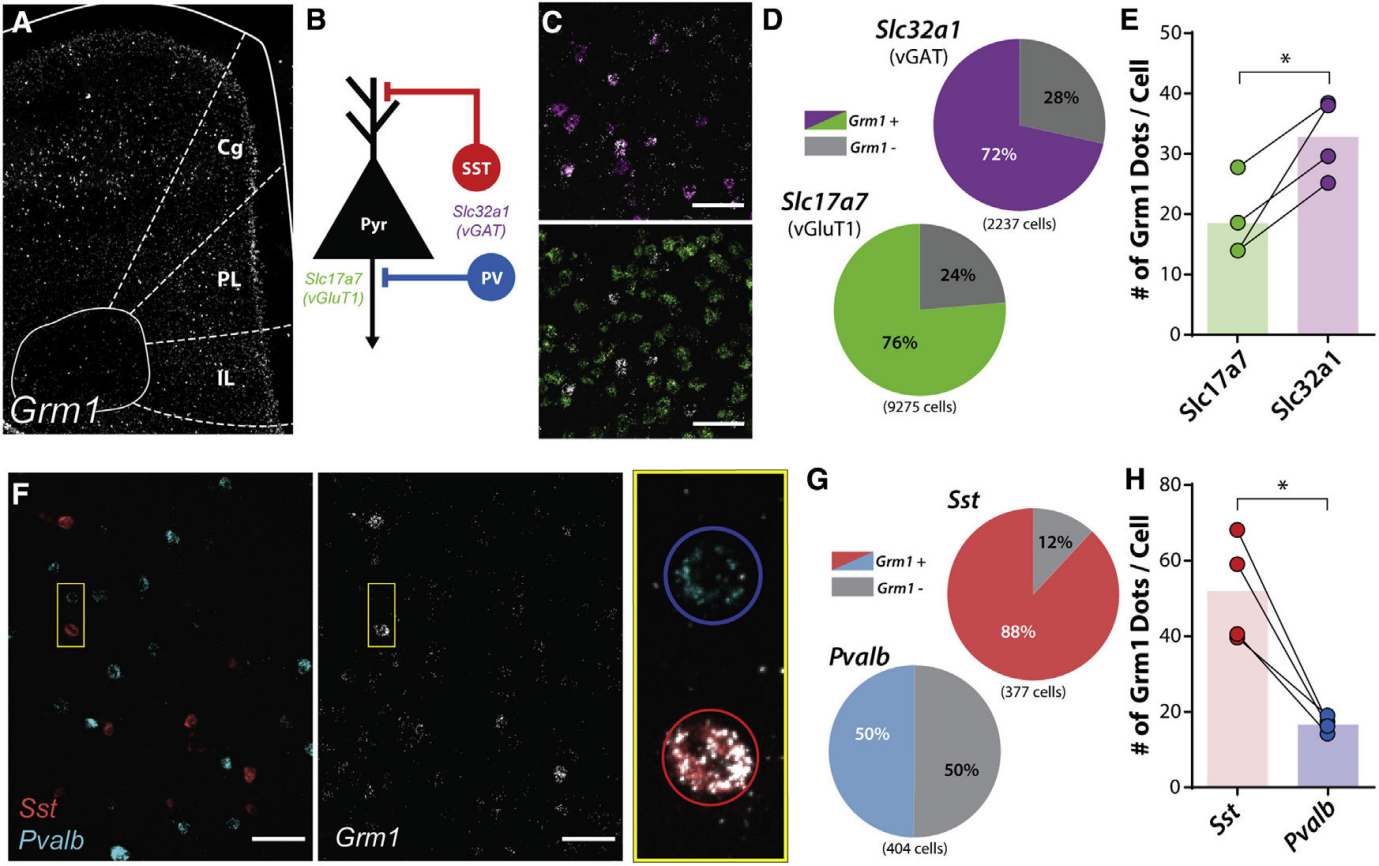
mGlu_1_ expression is enriched in prelimbic cortical somatostatin interneurons (A) *In situ* hybridization of *Grm1* mRNA in a coronal slice containing the mouse mPFC with regions outlined in white. Cg, cingulate; PL, prelimbic; IL, infralimbic. (B) Simplified schematic of cortical microcircuitry with mRNA markers for each neuronal population including glutamatergic pyramidal neurons (Pyr; *Slc17a7*, mRNA for vGluT1) and GABAergic interneurons (*Slc32a1*, mRNA for vGAT), consisting of somatostatin (SST)-positive (Sst), and parvalbumin (PV)-positive interneurons (*Pvalb*). (C) Representative images of deep layer V PL PFC. (Top) Colocalization of *Slc32a1* (magenta) and *Grm1* (white) mRNA. (Bottom) Colocalization of *Slc17a7* (green) and *Grm1* mRNA. Scale bars, 50 μm. (D) Percentage of *Grm1* mRNA colocalization with *Slc32a1*- and *Slc17a7*-positive cells. A greater proportion of *Slc17a7*-positive cells were *Grm1*-positive compared to *Slc32a1*-positive cells (*Grm1/Slc17a7*-posiitve cells, 7,079/9,275; *Grm1/Slc32a1*-positive cells, 1,600/2,237; two-sided Fisher’s exact test, p < 0.0001). (E) Quantification of number of *Grm1* mRNA puncta (dots) per *Slc32a1*- and *Slc17a7*-positive cell (two-tailed paired t test, p = 0.023, N = 4 mice). (F) Representative images of deep layer V PL PFC. (Left) *Sst*-positive (red) or *Pvalb*-positive (cyan) cells. (Middle) Same image displaying *Grm1* mRNA (white). (Right) Magnification of pair of cells outlined in yellow box with *Grm1, Sst, Pvalb* mRNA overlaid. SST-positive neuron outlined in red circle; PV-positive neuron outlined in blue circle. Scale bars, 50μm. (G) Percentage of *Grm1* mRNA colocalization with *Sst*- and *Pvalb*-positive cells. A greater proportion of *Sst*-positive cells was *Grm1*-positive compared to *Pvalb*-positive cells (*Grm1/Sst*-positive cells, 332/377; *Grm1/Pvalb*-positive cells, 201/404; two-sided Fisher’s exact test, p < 0.0001). (H) Quantification of number of *Grm1* mRNA puncta (dots) per *Sst*- and *Pvalb*-positive cell (two-tailed paired t test, p = 0.015, N = 4 mice). *p < 0.05. See also Figure S1.

While mGlu_1_ is expressed in SST-INs in the hippocampus and various cortical regions, the relative distribution of mGlu_1_ in the diverse neuronal populations in the rodent mPFC is unknown. We therefore used an *in situ* hybridization approach to visualize and colocalize mRNA encoding mGlu_1_, *Grm1*, throughout the mouse PL PFC (Figures 1A and 1B). *Grm1* mRNA was detectable in most putative glutamatergic vGluT1 (*S/c17a7*)-positive (76%, 7,079/9,275 cells) and GABAergic vGAT (*Slc32a1*)-positive (72%, 1,600/2,237) cells in the PL PFC (Figures 1C and 1D) although *Grm1* expression was significantly enriched in vGAT-positive compared with vGluT1-positive neurons (Figure 1E).

We then determined the GABAergic interneuron subtypes that express *Grm1*, focusing our subsequent experiments on SST- and PV-INs, which account for ~70% of all GABAergic neurons in the PL PFC and are the predominant subtypes within the deep cortical layers (Figure 1B). A greater proportion of SST-positive cells expressed *Grm1* (88%, 332/377) compared to PV-positive cells (50%, 201/404) (Figures 1F and 1G). Furthermore, when we compared relative expression within *Grm1*-positive cells, we observed a marked enrichment of *Grm1* mRNA puncta in SST-positive cells compared to PV-positive cells (Figure 1H). There were no differences in the distribution of *Grm1*-positive SST or PV cells or in the expression of *Grm1* across the different layers of the PL PFC (Figure S1). Taken together, these data demonstrate that mGlu_1_ expression is enriched in SST-INs in the rodent PL PFC.

### mGlu_1_ activation enhances SST-IN output in the PL PFC

To assess the functional consequences of mGlu_1_ expression differences between PL PFC SST- and PV-INs, we generated mice that express tdTomato in SST- or PV-INs, respectively. tdTomato-positive neurons from SST-Cre::Ai9 and PV-Cre::Ai9 mice were distributed throughout the mPFC (Figure S2A) and exhibited functional properties consistent with SST- and PV-INs, respectively (Figures S2B and S2C) (Joffe et al., 2020; Markram et al., 2004; McGarry and Carter, 2016).

**Figure 2.**
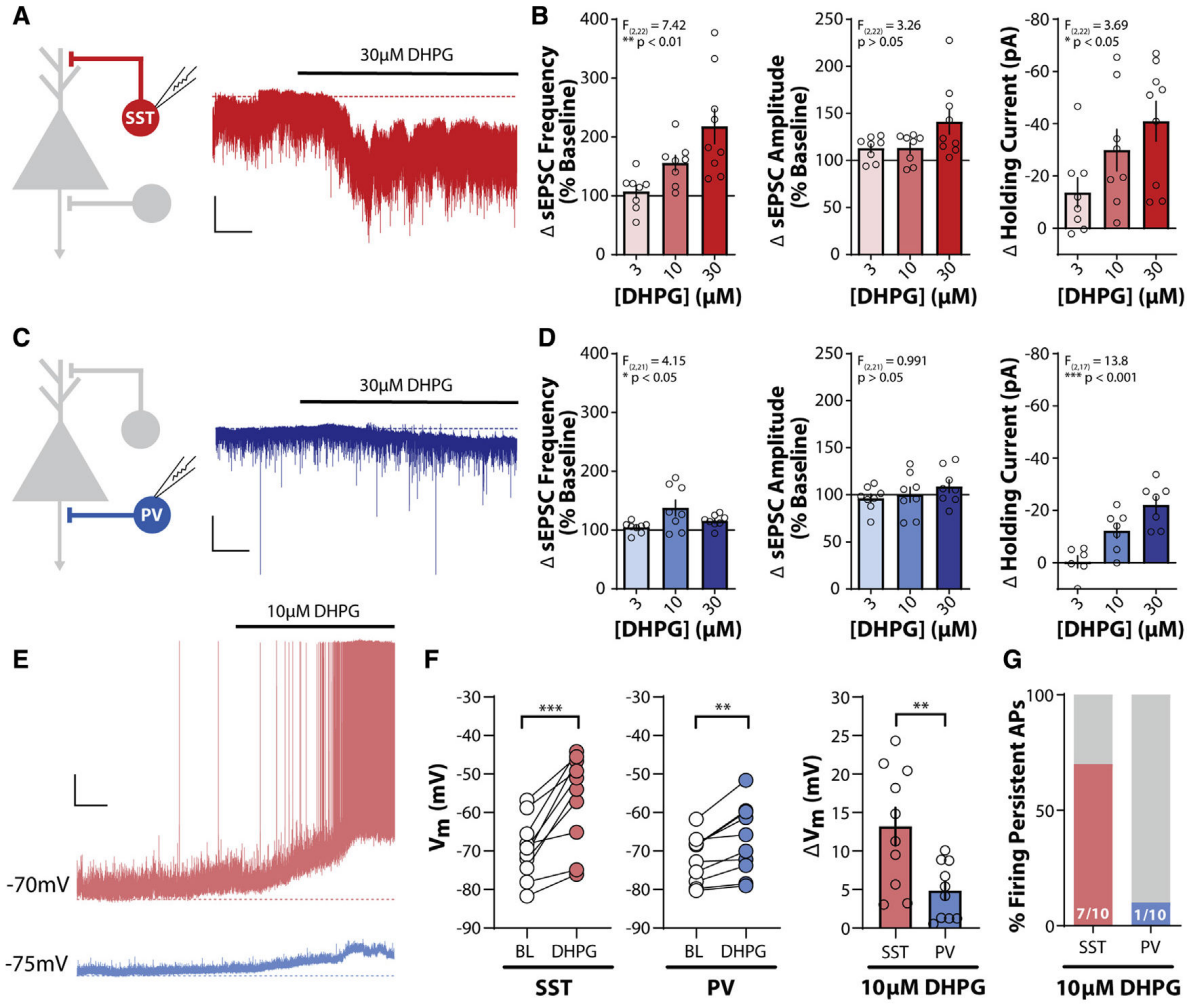
mGlu_1_ activation enhances SST interneuron output in the PL PFC (A) (Left) Schematic depicting whole-cell recording of SST-INs. (Right) Sample trace of voltage-clamp recording of an SST-IN in response to bath application of the mGlu_1/5_ agonist DHPG (30 μM), represented by bold black line above trace. All experiments were conducted in the constant presence of the mGlu_5_ negative allosteric modulator MTEP (3 μM). Dashed red line represents baseline holding current. Scale bars, 50 pA, 50 s. (B) DHPG induces a concentration-dependent increase in sEPSC frequency (one-way ANOVA main effect of DHPG concentration, F_(2,22)_ = 7.42, p = 0.0034), sEPSC amplitude (one-way ANOVA main effect of DHPG concentration, F_(2,22)_ = 3.26, p = 0.058), and holding current, I_h_ (one-way ANOVA main effect of DHPG concentration, F_(2,22)_ = 3.69, p = 0.042) in SST-INs (n/N = 8/5, 8/3, and 9/7 cells/mouse for 3, 10, and 30 μM DHPG). (C) (Left) Schematic depicting whole-cell recording of PV-IN. (Right) Sample trace of voltage-clamp recording of a PV-IN as in (A). Dashed blue line represents baseline holding current. Scale bars, 50 pA, 50 s. (D) Change in sEPSC frequency (one-way ANOVA main effect of DHPG concentration, F(2,21) = 4.15, p = 0.030), sEPSC amplitude (one-way ANOVA main effect of DHPG concentration, F_(2,21)_ = 0.991, p = 0.38), and holding current (one-way ANOVA main effect of DHPG concentration, F_(2,17)_ = 13.80, p = 0.0003) in response to bath application of DHPG in PV-INs (n/N = 8/4, 8/4, and 8/5 for 3, 10, and 30 μM DHPG effects on sEPSCs. n/N = 6/4, 7/4, and 7/5 for 3, 10, and 30 μM DHPG effects on I_h_). (E) Sample traces of current-clamp recordings from an SST-IN (red) and PV-IN (blue) in response to bath application of 10 μM DHPG. Dashed lines represent baseline membrane potential. Scale bars, 10 mV, 1 min. (F) DHPG (10 μM) depolarizes the membrane potential in SST-INs (left) and PV-INs (middle) (two-tailed paired t test, SST: p = 0.0005, PV: p = 0.0024; n/N = 10/4 per cell type). (Right) Greater depolarization was observed in SST-INs relative to PV-INs (two-tailed Student’s t test, p = 0.0066, n/N = 10/4 per cell type). (G) A greater percentage of SST-INs fire persistent action potentials in response to 10 μM DHPG. Number of cells responding/total cells recorded denoted in each bar. Two-sided Fisher’s exact test, p = 0.020. **p < 0.01, ***p < 0.001. Data are represented as mean ± SEM. See also Figure S2.

To pharmacologically isolate mGlu_1_ activation, we bath applied the group I mGlu receptor agonist DHPG in the constant presence of the mGlu_5_ negative allosteric modulator (NAM) MTEP while recording from PL PFC SST- or PV-INs (Figures 2A and 2C). In SST-INs, we observed a concentration-dependent increase in spontaneous excitatory postsynaptic current (sEPSC) frequency and a depolarizing change in the holding current, with no significant change in sEPSC amplitude (Figure 2B). In PV-INs, we observed similar concentration-dependent effects on sEPSC frequency and holding current but with a reduced magnitude compared to those in SST-INs (Figure 2D). At 30 mM DHPG, 100% of SST-INs (9/9) and PV-INs (7/7) exhibited depolarizing changes in holding current.

Based on the functional differences between SST- and PV-INs (Figure S2), we hypothesized that mGlu_1_ activation would have a greater effect on the output of SST-INs. To test this, we recorded from interneurons in current-clamp configuration. While mGlu_1_ activation depolarized both interneuron populations, SST-INs exhibited greater depolarization compared to PV-INs (Figure 2F). mGlu_1_ activation induced persistent action potential (AP) firing in most SST-INs (7/10), whereas only 1 of 10 PV-INs depolarized sufficiently to persistently fire APs (Figure 2G). Furthermore, a maximal concentration of 30 mM DHPG was insufficient to induce AP firing in PV-INs (Figure S4C). Altogether, these data demonstrate that activation of mGlu_1_ preferentially increases the output of PL PFC SST-INs.

### mGlu_1_, activation increases inhibitory transmission onto layer V pyramidal neurons via actions on SST-INs

**Figure 3.**
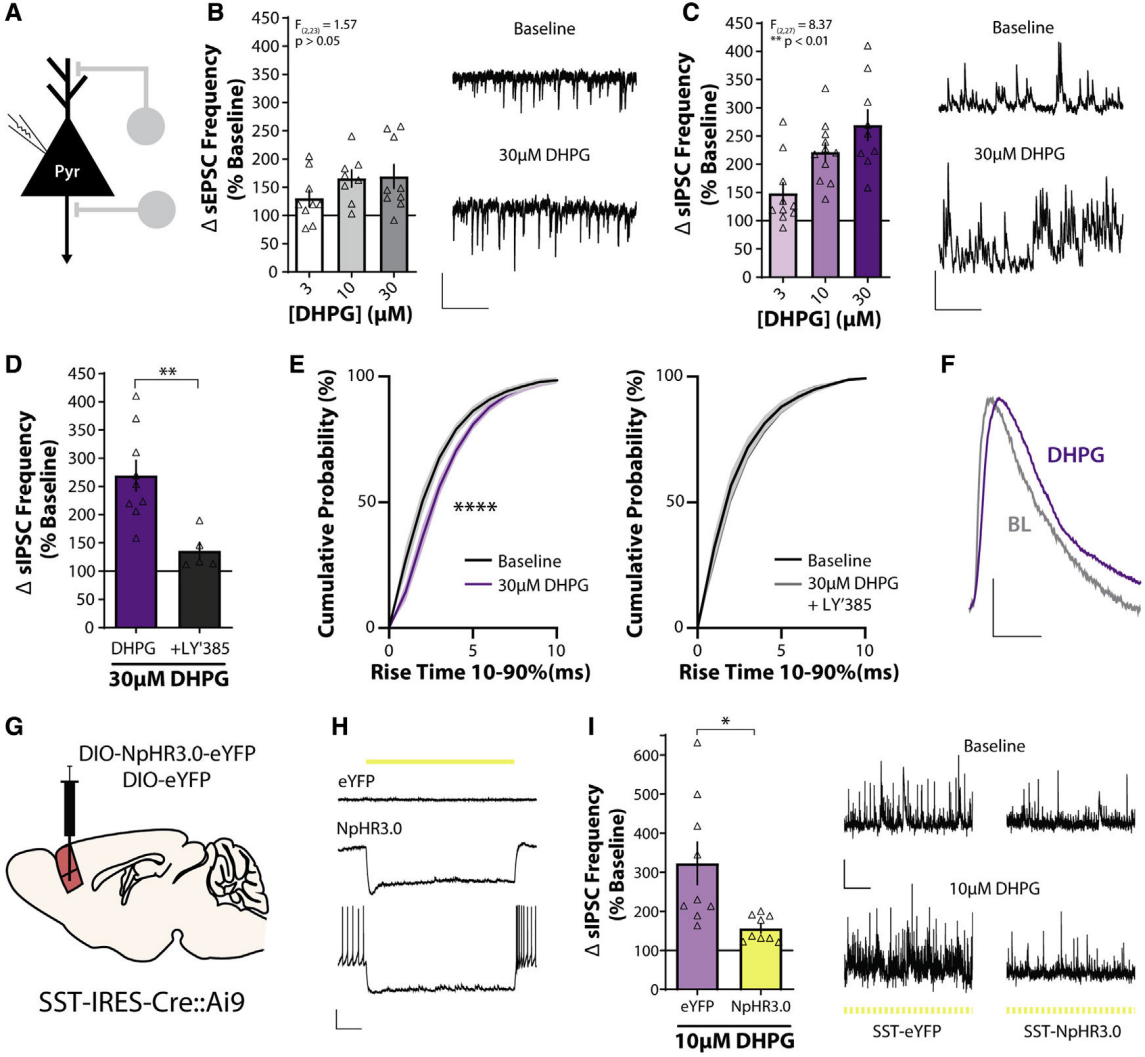
SST interneurons mediate mGlu_1_ receptor activation-induced increases in PL PFC inhibitory transmission (A) Schematic depicting whole-cell recording of layer V pyramidal neuron. (B) (Left) Change in sEPSC frequency in layer V pyramidal neurons in response to bath application of DHPG expressed as a percentage of baseline (one-way ANOVA main effect of DHPG concentration, F_(2,23)_ = 1.57, p = 0.23, n/N = 9/4, 8/6, and 9/6 cells/mouse for 3, 10, and 30 μM DHPG). (Right) Sample traces showing sEPSCs recorded in voltage clamp at −70 mV during baseline and in the presence of 30 μM DHPG. All experiments were conducted in the constant presence of the mGlu5 negative allosteric modulator MTEP (3 μM). Scale bars, 20 pA, 500 ms. (C) (Left) DHPG induces a concentration-dependent increase in sIPSC frequency in response to bath application of DHPG (one-way ANOVA main effect of DHPG concentration, F_(2,27)_ = 8.37, p = 0.0015, n/N = 10/8, 11/9, and 9/6 for 3, 10, and 30 μM DHPG). (Right) Sample traces showing sIPSCs recorded in voltage clamp at +10 mV during baseline and in the presence of 30 μM DHPG. Scale bars, 50 pA, 500 ms. (D) The mGlu_1_ antagonist LY367385 (100 μM) blocks the increase in sIPSC frequency induced by 30 μM DHPG (two-tailed unpaired Student’s t test, p = 0.0047, n/N = 9/6 for DHPG, 5/2 for +LY′385). (E) Cumulative probability plots of sIPSC rise time during baseline and after bath application of 30 μM DHPG alone (left; two-way repeated-measures ANOVA, main effect of drug, F_(1,88)_ = 67.5, p < 0.0001) or in the presence of 100 μMLY367385 (right; two-way repeated measures ANOVA, main effect of drug, F_(1,44)_ = 2.65, p = 0.11). (F) Scaled: average sIPSCs at baseline (BL) and after 30 μM DHPG from representative traces in (C). Scale bars, scaled amplitude, 10 ms. (G) Schematic depicting approach for viral-mediated expression of NpHR3.0-EYFP or EYFP in mPFC SST-INs. (H) Representative current-clamp recordings from control EYFP-infected (top) or NpHR3.0-EYFP-infected (middle and bottom) SST-INs. Delivery of 565-nm light is depicted by the yellow line. (I) (Left) 565-nm light (100-ms duration, 5 Hz) blocks the DHPG-induced increase in sIPSC frequency in NpHR3.0-expressing slices, but not EYFP-expressing slices (two-tailed unpaired Student’s t test with Welch’s correction, p = 0.015, n/N = 9/3 for EYFP, 9/5 for NpHR3.0). (Right) Sample traces showing sIPSCs during baseline and 10 μM DHPG in pulsed yellow light in control and NpHR3.0-infected slices. Scale bars, 50 pA, 2 s. *p < 0.05, **p < 0.01. Data are represented as mean ± SEM. See also Figure S3.

Cortical interneurons are critical for the modulation and synchronization of glutamatergic pyramidal neurons. We next focused on the functional effects of mGlu_1_ activation on layer V pyramidal neurons (Figure 3A), which constitute a large proportion of mPFC output neurons (Harris and Shepherd, 2015). mGlu_1_ activation caused a small increase in sEPSC frequency onto layer V pyramidal cells, although this was not concentration-dependent (Figure 3B). We then recorded GABAergic spontaneous inhibitory postsynaptic currents (sIPSCs) and found that activation of mGlu_1_ resulted in a robust, concentration-dependent increase in sIPSC frequency onto layer V pyramidal neurons (Figure 3C). sIPSC amplitude was not affected (3 mM, 112% ± 12.7%; 10 μM, 125% ± 17.0%; 30 μM, 126% ± 10.7% baseline; one-way ANOVA main effect of DHPG concentration, F_(2,27)_ = 0.311, p = 0.74), consistent with a presynaptic mechanism involving mGlu_1_ receptors on SST-INs. The selective mGlu_1_ antagonist LY367385 blocked the effect of 30μM DHPG on sIPSC frequency, confirming an mGlu_1_-dependent mechanism (Figure 3D). These data indicate that mGlu_1_ activation preferentially increases inhibitory transmission onto layer V pyramidal cells, consistent with previous findings (Sun and Neugebauer, 2011).

SST-INs preferentially synapse onto the distal dendrites of layer V pyramidal neurons (Rudy et al., 2011), and IPSCs generated from synapses farther away from the somatic recording site exhibit slower kinetics than do those originating from perisomatic synapses (Magee, 2000; McGarry and Carter, 2016). DHPG application prolonged the sIPSC rise time and this effect was blocked by LY367385 (Figure 3E), consistent with mGlu_1_ activation augmenting SST-IN output. Next, we directly tested whether SST-IN activity is necessary for the mGlu_1_-mediated increase of inhibitory drive onto pyramidal cells. We injected an adeno-associated virus expressing a Cre-dependent inhibitory chloride pump halorhodopsin (NpHR3.0) into the PL PFC of SST-Cre::Ai9 mice (Figure 3G) and observed NpHR3.0-positive neurons throughout the PL PFC colocalized with tdTomato (Figure S3A). Brief pulses of 565-nm light selectively hyperpolarized NpHR3.0/tdTomato-positive SST-INs and blocked AP firing (Figure 3H). We then recorded sIPSCs from nearby layer V pyramidal neurons, activated mGlu_1_, and inhibited SST-INs with yellow light (Figure S3B). In control, EYFP-expressing slices, we replicated a robust increase in sIPSC frequency onto layer V pyramidal cells (Figure 3I). In contrast, this effect was significantly attenuated in NpHR3.0-expressing slices (Figure 3I). DHPG also increased sIPSC rise time in EYFP- but not in NpHR3.0-infected slices (Figure S3D). These data demonstrate that the mGlu_1_-mediated increase of GABAergic transmission onto PL PFC pyramidal neurons preferentially involves augmented SST-IN output, although network-level effects may also contribute.

### The mGlu_1_ PAM VU6004909 shifts PL PFC I/E balance toward inhibition

**Figure 4.**
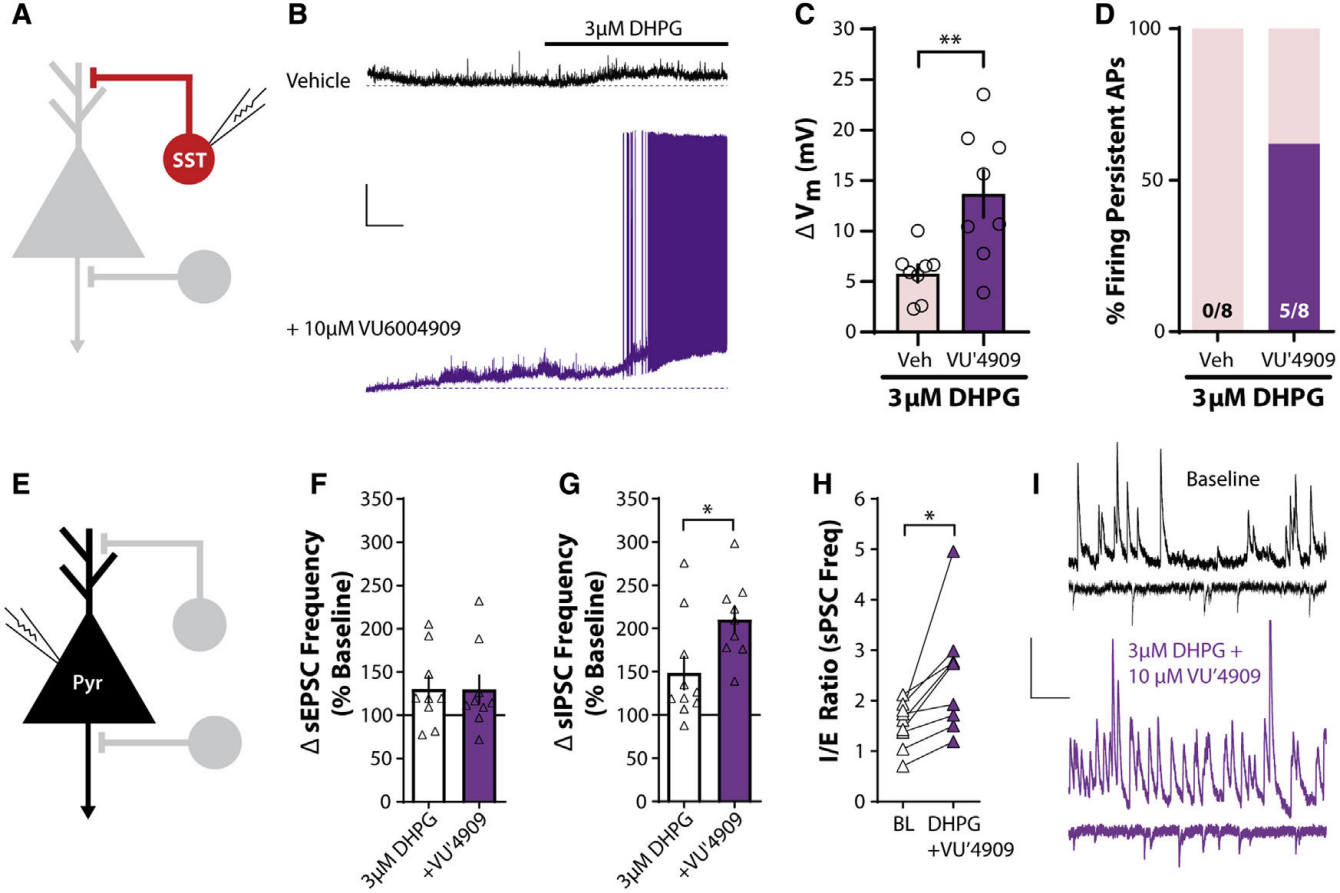
The mGlu_1_ PAM VU6004909 shifts I/E balance toward inhibition (A) Schematic depicting whole-cell recording of SST-INs. (B) Sample traces of current-clamp recordings from SST-INs in response to bath application of 3 μM DHPG in the presence of vehicle (black) or 10 μMVU6004909 (purple). Dashed lines represent baseline membrane potential. All experiments were conducted in the constant presence of the mGlu_5_ negative allosteric modulator MTEP (3 μM). Scale bars, 10 mV, 1 min. (C) VU6004909 (10 μM) increases the SST-IN depolarization in response to 3 μM DHPG (two-tailed unpaired Student’s t test, p = 0.0067, n/N = 8/4 cells/mouse for Veh, 8/4 for VU′4909). (D) A greater percentage of cells fire persistent action potentials in response to 3 μM DHPG in the presence of VU6004909. Number of cells responding/total cells recorded is denoted in each bar. Two-sided Fisher’s exact test, p = 0.026. (E) Schematic depicting whole-cell recording of layer V pyramidal neurons. (F) No difference in sEPSC frequency in layer V pyramidal neurons in response to bath application of 3 μM DHPG with and without 10 μM VU6004909 (two-tailed unpaired Student’s t test, p = 0.98, n/N = 9/4 for 3 μM DHPG, 9/4 for +VU′4909). (G) VU6004909 (10 μM) potentiates the increase in sIPSC frequency in response to bath application of 3 μM DHPG (two-tailed unpaired Student’s t test, p = 0.023, n/N = 10/8 for 3 μM DHPG, 9/4 for +VU′4909). (H) Ratio of sIPSC to sEPSC frequency (I/E ratio) in the same layer V pyramidal neuron during baseline and in the presence of 3 μM DHPG and 10 μM VU6004909. DHPG + VU6004909 significantly increased the I/E ratio (two-tailed paired t test, p = 0.012, n/N = 9/4). (I) Sample traces of sIPSCs and sEPSCs in layer V pyramidal neurons during baseline and in the presence of 3 μM DHPG and 10 μM VU6004909. Scale bars, 20 pA, 250 ms. *p < 0.05, **p < 0.01. Data are represented as mean ± SEM. See also Figure S4.

Based on our previous results and to test the hypothesis that potentiating mGlu_1_ function selectively enhances PL PFC inhibition, we took advantage of the recently developed mGlu_1_ PAM tool compound VU6004909 that exhibits excellent subtype selectivity (mGlu_1_ PAM half-maximal effective concentration [EC_50_] 25 nM; other mGlu receptor subtypes >10 μM) and pharmacokinetic properties for *in vivo* use (Garcia-Barrantes et al., 2016). To test whether the mGlu_1_ PAM VU6004909 potentiates inhibition in native tissue, we again recorded from SST-INs (Figure 4A). Pretreatment of slices with the mGlu_1_ PAM VU6004909 (10 μM) significantly enhanced SST-IN depolarization in response to a threshold concentration of DHPG (3 μM) (Figures 4B and 4C). VU6004909 also increased the proportion of SST-INs that fired persistent APs in response to DHPG (Figure 4D). In contrast, VU6004909 did not elicit APs in PV-INs in response to threshold or maximal concentrations of DHPG (Figure S4). These data indicate that an mGlu_1_ PAM preferentially enhances the output of SST-INs in the PL PFC.

In recordings from layer V pyramidal neurons (Figure 4E), VU6004909 had no effect on sEPSC frequency (Figure 4F) but significantly potentiated the effect of 3 μM DHPG on sIPSC frequency (Figure 4G). VU6004909 also enhanced sIPSC rise time, consistent with augmented SST-IN-mediated inhibition (Figure S4G). Furthermore, we compared the ratio of sIPSC to sEPSC frequency at baseline and after drug wash-on within the same pyramidal neurons. The combination of 3 μM DHPG and VU6004909 significantly increased the inhibitory/excitatory (I/E) ratio (Figure 4H). These data demonstrate that an mGlu_1_ PAM preferentially potentiates PL PFC inhibitory transmission and suggest that VU6004909 could rescue I/E imbalance *in vivo*.

### mGlu_1_ potentiation ameliorates cortical hyperactivity induced by NMDA receptor hypofunction

To examine the efficacy of an mGlu_1_ PAM *in vivo*, we employed an NMDA receptor hypofunction model of schizophrenia. NMDA receptor antagonists, such as MK-801, induce schizophrenia-like symptoms including cognitive deficits in healthy individuals (Javitt and Zukin, 1991) and can precipitate symptoms in patients with schizophrenia. Converging with the GABAergic dysfunction hypothesis, administration of the NMDA receptor antagonist MK-801 in rodents increases mPFC pyramidal neuron firing while decreasing the activity of PV- and SST-INs (Ali et al., 2020; Homayoun and Moghaddam, 2007). These findings suggest that NMDA receptor antagonism induces disinhibition and disrupts cortical I/E balance, modeling schizophrenia-like functional deficits in inhibitory transmission (Cohen et al., 2015).

**Figure 5.**
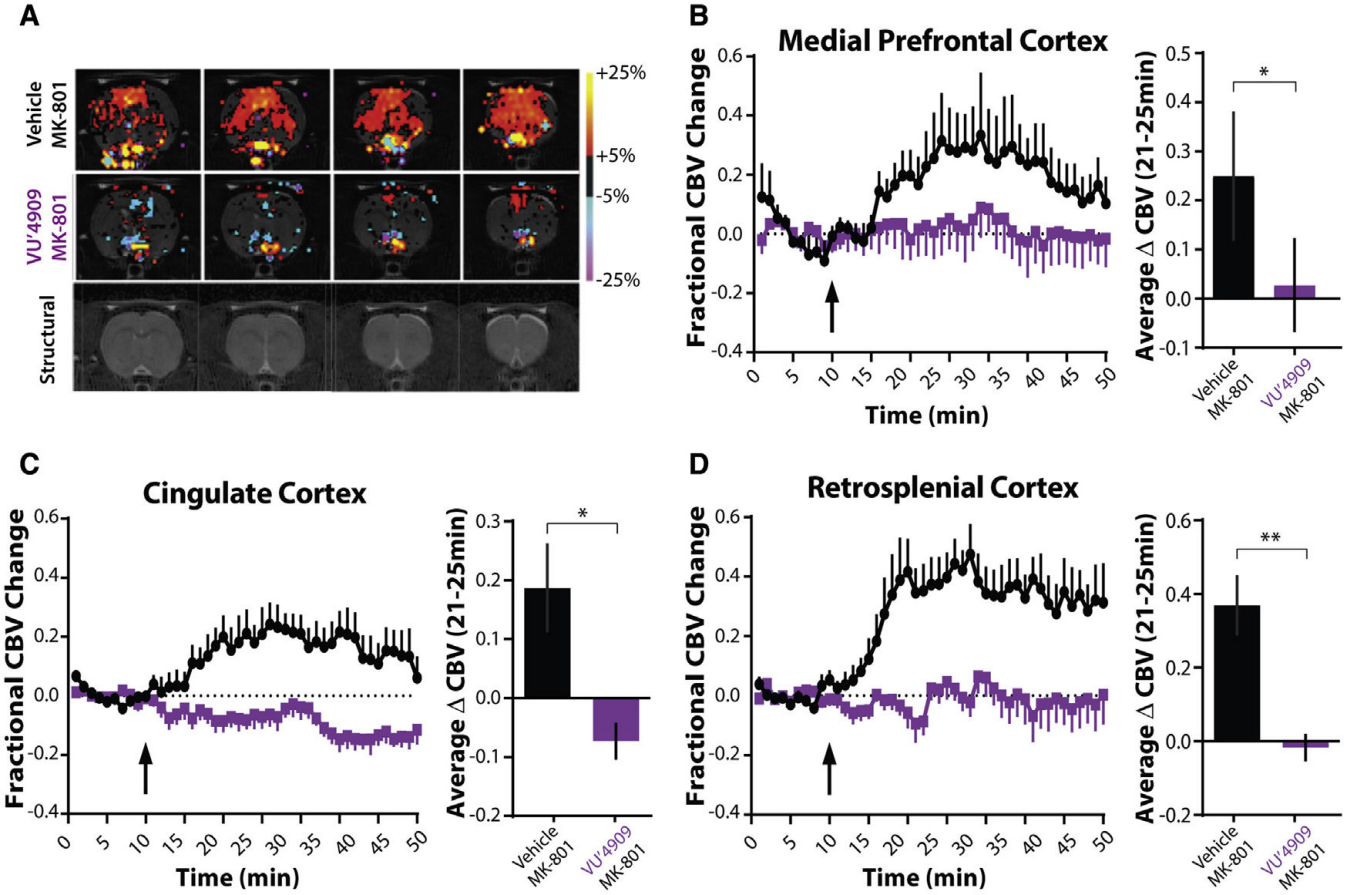
mGlu_1_ potentiation reverses NMDA receptor antagonist-induced cortical hyperactivity (A) Representative cerebral blood volume (CBV) and structural T2-weighted MRI template images of male Sprague-Dawley rats anesthetized and then treated with vehicle or 60 mg/kg VU6004909 (i.p., 10% Tween 80) and then 0.3 mg/kg MK-801 (s.c., 0.9% saline). In the group activation maps, the red to yellow bar range represents increased CBV, indicating increased neuronal activity, while the blue to purple color bar range represents decreased CBV, indicating decreased neuronal activity compared to the pre-drug baseline period. (B–D) Time courses and bar graphs of CBV changes after MK-801 injection (arrow) in rats pretreated with vehicle or VU6004909 from (B) medial prefrontal cortex (mPFC), (C) cingulate cortex (Cg), or (D) retrosplenial cortex (RSC). Time courses show fractional changes in CBV (ΔCBV(t)/CBV_o_). For bar graphs, fractional CBV values were averaged between 21 and 25 min for each animal. VU6004909 blocks the MK-801-induced increase in fractional CBV across multiple cortical areas (two-tailed Mann-Whitney test; mPFC, p = 0.041; Cg, p = 0.015; RSC, p = 0.0043; N = 6 rats per group). *p < 0.05, **p < 0.01. Data are represented as mean ± SEM. See also Figure S5.

Accompanying the behavioral effects, NMDA receptor antagonism precipitates a widespread increase in brain activity in healthy volunteers, particularly in the dlPFC (Doyle et al., 2013). Moreover, increased cortical hyperactivity is also observed in people with schizophrenia during working memory tasks (Manoach et al., 1999). To model this effect in rodents in a translationally relevant manner, we used pharmacological magnetic resonance imaging (phMRI) to assess the physiological effects of an mGlu_1_ PAM on MK-801-induced cortical hyperactivity in anesthetized rats. We measured cerebral blood volume (CBV) changes that indirectly reflect alterations in neural activity and assessed cortical, hippocampal, striatal, and thalamic areas (Figures 5A and S5A). Changes in electrically measured neuronal activity positively correlate with changes in hemodynamic processes including CBV (Gore, 2003). Thus, enhanced regional CBV, which could indicate hyperexcitability and reduced inhibition (Havlicek et al., 2017), can be interpreted as an indirect proxy of increased neuronal activity.

In line with previous phMRI findings with an NMDA receptor antagonist (Hackler et al., 2010), injection of a behaviorally active dose of MK-801 (0.3 mg/kg, subcutaneously [s.c.]) led to sustained increases in CBV across cortical areas including the mPFC (Figure 5B), cingulate cortex (Cg) (Figure 5C), and retrosplenial cortex (RSC) (Figure 5D) as well as the dorsal and ventral striatum, thalamic nuclei, and hippocampus (Figures S5B-S5H). Pre-treatment with VU6004909 (60 mg/kg, intraperitoneally [i.p.]) significantly reversed MK801-induced cortical and subcortical hyperactivity. These reversals were significant in the mPFC, Cg, and RSC (Figures 5B-5D) as well as in the nucleus accumbens (Figure S5D) but not in the hippocampus (Figure S5H). VU6004909-mediated effects on the MK-801 response in the motor circuit (i.e., motor cortex, substantia nigra, caudate-putamen) were not significant (Figures S5B, S5C, and S5G), potentially due to a dampening of brain responses by the anesthesia necessary to immobilize animals during imaging. No effect of VU6004909 was observed in thalamic regions (Figures S5E and S5F). Nonetheless, the cortical data demonstrate that an mGlu_1_ PAM can reverse cortical hyperactivity *in vivo* and are consistent with mGlu_1_ PAMs shifting cortical I/E balance in favor of inhibition.

### The mGlU_1_ PAM VU6004909 reverses MK-801-induced deficits in spatial working memory

**Figure 6.**
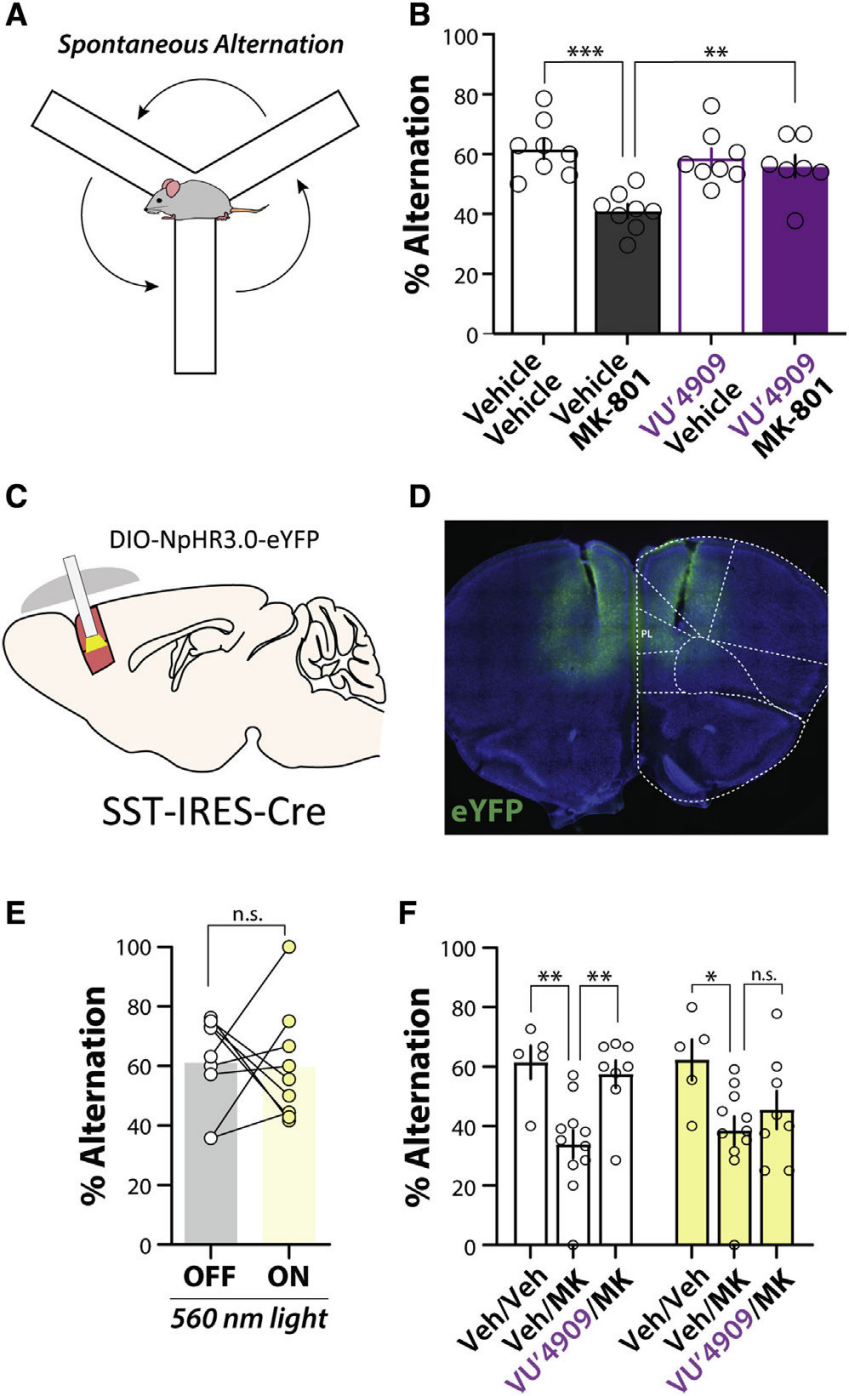
The mGlu_1_ PAM VU6004909 reverses an MK-801-induced working memory deficit via actions on PL PFC SST interneurons (A) Schematic of spontaneous alternation in the Y-maze. (B) Average % spontaneous alternation in mice pretreated with vehicle or 60 mg/kg VU6004909 (i.p., 10% Tween 80) prior to administration of vehicle or 0.18 mg/kg MK-801 (i.p., 0.9% saline) 20 min before behavioral test. MK-801 impairs spontaneous alternation and this deficit is reversed by VU6004909 (one-way ANOVA main effect, F_(3,27)_ = 8.99, p = 0.0003; post hoc Bonferroni’s test: vehicle/vehicle versus vehicle/MK-801, p = 0.0002; vehicle/MK-801 versus VU′4909/MK-801, p = 0.0077; vehicle/vehicle versus VU′4909/vehicle, p = 1.00; N = 8 mice for vehicle/vehicle, 8 for vehicle/MK-801, 8 for VU′4909/vehicle, and 7 for VU′4909/MK-801). (C) Schematic depicting strategy to optogenetically inhibit SST-INs *in vivo*. (D) Representative image of bilateral NpHR3.0-EYFP expression throughout the mPFC and bilateral fiber tracts terminating in the PL PFC. (E) No effect of PL PFC SST-IN inhibition on spontaneous alternation in the Y-maze. SST-NpHR mice underwent an 8-min trial with 4 min of light ON and 4 min of light OFF, randomized to either receive ON first or OFF first (see Figure S6). No effect of light order was observed; therefore, data are presented as pooled OFF and ON trials (two-tailed paired t test, p = 0.88, N = 9). (F) Inhibiting PL PFC SST-INs blocks the ability of VU6004909 to reverse MK-801-induced deficits in the Y-maze. Effect is shown of 560-nm light on Y-maze performance in mice dosed with vehicle, 0.18 mg/kg MK-801 (i.p., 0.9% saline), or 60 mg/kg VU6004909 (i.p., 10% Tween 80) pretreated before MK-801. White bars represent performance during light OFF trials, and yellow bars represent performance during light ON trials (two-way repeated measures ANOVA, main effect of drug, F_(2,21)_ = 9.33, p = 0.0013; post hoc Bonferroni’s multiple comparisons test: OFF trials, vehicle/vehicle versus vehicle/MK-801, p = 0.0037; vehicle/MK-801 versus VU′4909/MK-801, p = 0.0039; ON trials, vehicle/vehicle versus vehicle/MK-801, p = 0.013; vehicle/MK-801 versus VU′4909/MK-801, p = 0.67; N = 5 for vehicle/vehicle, 11 for vehicle/MK-801, and 8 for VU′4909/MK-801). *p < 0.05, **p < 0.01. Data are represented as mean ± SEM. See also Figure S6.

In addition to modeling physiological dysfunction in schizophrenia, systemic administration of NMDA receptor antagonists induces a range of behavioral deficits relevant to the cognitive symptoms of schizophrenia (Jones et al., 2011). Therefore, we determined whether the mGlu_1_ PAM VU6004909 has efficacy in reversing an MK-801-induced deficit in mPFC-dependent cognitive function. We assessed spatial working memory in mice by monitoring spontaneous alternation in the Y-maze (Figure 6A), a task requiring mPFC interneuron function (Murray et al., 2015). We observed a significant deficit in spontaneous alternation induced by MK-801 (0.18 mg/kg, i.p.) that was rescued by pretreatment with VU6004909 (60 mg/kg, i.p.) (Figure 6B). Importantly, this was not accompanied by a reduction in locomotion as assessed by total arm entries (Figure S6A), demonstrating a procognitive effect of the mGlu_1_ PAM. Similarly, the structurally distinct mGlu_1_ PAM Ro-07-11401 (Vieira et al., 2009) (30 mg/kg, i.p.) reversed MK-801-induced deficits in spontaneous alternation (Figures S6B and S6C). Taken together, these data demonstrate that selective mGlu_1_ potentiation *in vivo* can reverse cognitive deficits induced by an NMDA receptor antagonist model of cortical disinhibition.

### PL PFC SST-IN activity is necessary for the mGlu_1_ PAM rescue of spatial working memory deficits

Lastly, we set out to determine whether the procognitive effects of an mGlu_1_ PAM require activation of PL PFC SST-INs. We used an optogenetic approach, bilaterally expressing NpHR3.0 in mPFC SST-INs and implanting fiber optic cannulae above the PL PFC to activate NpHR3.0 *in vivo* (Figures 6C and 6D). We then used the Y-maze task to test the hypothesis that PL PFC SST-IN activity is required for mGlu_1_ PAM efficacy. We first tested whether PL PFC SST-IN activity was necessary for spatial working memory in the Y-maze with mice performing half of the task with the light off and the other half with the light on (Figure S6E), using the samelight stimulation parameters that we previously characterized in slices (Figure 3). Although mice decreased the number of arm entries as they habituated to the maze, this did not affect performance during control trials where mice that were connected to the fiber did not receive light (Figure S6F). Consistent with ablation studies (Murray et al., 2015), optogenetic inhibition of PL PFC SST-INs had no effect on spontaneous alternation performance (Figure 6E).

With no effect on general alternation behavior, we were able to test the hypothesis that PL PFC SST-INs are required for mGlu_1_ PAM efficacy. Mice were randomized to receive light on during the first or second half of the task (Figure S6E). In vehicle-treated mice, we observed no difference in performance depending on the order of NpHR3.0 stimulation; therefore, all data were pooled and presented as light-off versus light-on trials. Replicating our previous findings in the light-off condition, we observed a significant deficit in performance induced by MK-801 compared to vehicle-treated mice. This deficit was rescued by the mGlu_1_ PAM VU6004909 in control light-off conditions (Figure 6F). However, in the light-on condition when PL PFC SST-IN activity was inhibited, VU6004909 did not reverse MK-801-induced deficits (Figure 6F). These data provide a causal association between mGlu_1_ PAM procognitive efficacy and the activity of PL PFC SST-INs, consistent with our mechanistic data in brain slices (Figures 2, 3, and 4).

## DISCUSSION

In this study, we found that mGlu_1_ activation and potentiation enhances inhibitory neurotransmission in the PL PFC predominantly via actions on SST-INs. We demonstrate that mGlu_1_ PAMs can reverse cortical hyperactivity and have efficacy in reversing working memory deficits induced by NMDA receptor antagonism *in vivo*, an effect dependent on the function of PL PFC SST-INs. The current data demonstrate that mGlu_1_ PAMs reverse physiological and cognitive deficits in a schizophreniarelevant model and support their potential to provide procognitive efficacy for the treatment of schizophrenia.

Modulation of the GABAergic system has long been pursued as a therapeutic approach for schizophrenia (Guidotti et al., 2005; Lewis et al., 2004; Rudolph and Möhler, 2014; Xu and Wong, 2018). While GABA_A_ receptors have garnered attention (Ballard et al., 2009; Burke et al., 2018; Geffen et al., 2012; Gill et al., 2011; Lewis et al., 2008; Prevot et al., 2019), enhancing interneuron function has lagged due to a lack of druggable targets selective for these distinct neuronal populations. Our data identify mGlu_1_ as a target to preferentially increase mPFC SST-IN activity, providing an alternative means to rescue deficient inhibitory transmission in patients with schizophrenia. Our data also add to accumulating evidence suggesting that enhancing SST-IN activity can improve cognitive function broadly. Transmission between SST-INs and pyramidal neurons is predominantly via α_5_-containing GABA_A_ receptors (Schulz et al., 2018), and α_5_ PAMs have procognitive efficacy in preclinical models (Koh et al., 2013; Prevot et al., 2019). α7 nicotinic and M_1_ muscarinic acetylcholine receptor modulators also augment SST-IN function and have procognitive effects in preclinical models and in early clinical trials (Conley et al., 2019; Ghoshal et al., 2016; Grannan et al., 2016; Haam et al., 2018; Lawrence et al., 2006; Poorthuis et al., 2013; Tregellas and Wylie, 2019; Wohleb et al., 2016). While the contributions of SST-IN modulation to these behavioral effects are unclear, these findings and our current results provide strong evidence for further discovery efforts directed at modulating SST-INs.

It is intriguing that targeting mPFC SST-INs reverses schizophrenia-like deficits in light of abundant evidence of PV-IN dysfunction in schizophrenia (Behrens et al., 2007; Gonzalez-Burgos et al., 2015; Homayoun and Moghaddam, 2007; Lodge et al., 2009; Mukherjee et al., 2019). Our results might indicate that SST-INs are also impaired in NMDA receptor antagonist models. In adult rodents, the NMDA receptor contribution to excitatory transmission onto PV-INs is relatively weak compared to SST-INs (McGarry and Carter, 2016; Rotaru et al., 2011; Wang and Gao, 2009); thus, SST-INs may be more susceptible to NMDA receptor antagonism. Consistent with this, a recent study found that acute systemic ketamine reduced SST-IN activity and increased pyramidal neuron activity *in vivo* in the cingulate cortex (Ali et al., 2020). Furthermore, genetic reduction of NMDA receptors in pyramidal neurons impairs SST-pyramidal neuron inhibitory transmission (Chiu et al., 2018; Horn and Nicoll, 2018). Interestingly, we found no deficit in Y-maze performance upon optogenetic inhibition of PL PFC SST-INs, consistent with prior work demonstrating that ablation of mPFC SST-INs does not impair spontaneous alternation (Murray et al., 2015). In a delayed non-match to sample task, inhibiting mPFC SST-INs only impaired performance at long delays (Abbas et al., 2018) that animals likely do not experience during spontaneous alternation. Therefore, while mPFC SST-INs appear to be dispensable for baseline performance in the spontaneous alternation task, potentiating their activity can rescue the MK-801-induced behavioral deficit.

Several corroborative lines of evidence support that SST-IN dysfunction contributes to schizophrenia etiology. In cortical regions, SST-INs contribute to low-frequency theta and beta oscillations, which are abnormal in schizophrenia (Moran and Hong, 2011). *SST* mRNA is reduced in the dlPFC of patients with schizophrenia (Fung et al., 2010, 2014; Hashimoto et al., 2008) and may imply a clinical deficit. Supporting the translatability of our results, a recent study in human temporal cortex found that group I mGlu receptor activation increased the output of putative SST-INs and enhanced inhibition onto layer II/III pyramidal neurons (Kroon et al., 2019). This suggests that the functional effects of mGlu_1_ potentiation may be conserved between rodents and humans and that an mGlu_1_ PAM could engage similar circuitry in patients with schizophrenia. Importantly, SST-INs are a heterogenous population with subtypes exhibiting different electrophysiological and morphological characteristics and distinct functions across cortical layers (Gouwens et al., 2020; Yavorska and Wehr, 2016). We did not observe any clear difference in *Grm1* expression across PL PFC layers, but whether mGlu_1_ modulation affects specific SST-IN subtypes merits examination in future studies.

Although our *ex vivo* and *in vivo* results suggest that mGlu_1_ PAM efficacy is primarily mediated through an SST-IN mechanism in the mPFC, our data do not rule out the modulation of other populations. PV-INs may be required for mGlu_1_ PAM efficacy, similar to recent data demonstrating that the antidepressant-like efficacy of ketamine requires both SST- and PV-IN subtypes (Gerhard et al., 2020). Furthermore, there is a question of how augmenting SST-IN output would rescue PV-IN dysfunction (Gonzalez-Burgos et al., 2015). We found evidence of network-level mGlu_1_ activation such as a higher than expected number of PV-INs responding to mGlu_1_ agonism. Approximately half of PV-INs did not express *Grm1* mRNA while all recorded neurons depolarized in response to DHPG. A possible explanation is the existence of gap junction-mediated electrical coupling between interneurons (Fukuda and Kosaka, 2000; Hatch et al., 2017). Additionally, we did not test whether PV-INs are required for the *in vivo* efficacy of mGlu_1_ PAMs, and it is possible that the subtler effects of mGlu_1_ potentiation on PV-INs *ex vivo* result in a more pronounced effect *in vivo*. Other mPFC macrocircuits and microcircuits could also be modulated by mGlu_1_, as many excitatory PL PFC cells express *Grm1*, and mGlu_1_ activation increases excitatory drive onto pyramidal, SST, and PV neurons. Furthermore, inhibition of SST-INs in the PL cortex most likely explains our *in vivo* optogenetic results but it is possible some SST-INs in the infralimbic cortex may also have been inhibited and thus contribute to mGlu_1_ PAM efficacy. While our data suggest a significant role of PL SST-INs in mediating the effects of mGlu_1_ PAMs, we cannot rule out more complex mPFC network-level modulation that will be interesting to investigate in the future.

While we describe a circuit-level mechanism of mGlu_1_ PAM action, the molecular mechanisms underlying mGlu_1_-mediated enhancement of SST-IN function are unclear. mGlu_1_-mediated depolarization of SST-INs could involve closing of leak potassium channels or activation of TrpC channels, both of which have been shown to be downstream of mGlu_1_ in other regions (Gee et al., 2003; Mannaioni et al., 2001). Additionally, endogenous mGlu_1_ activation by high-frequency stimulation can elicit a slow EPSC in hippocampal and cerebellar neurons (Hartmann et al., 2011), but whether this occurs in mPFC SST-INs and the mechanism are unknown. Whether mGlu_1_ receptors in other brain regions and other cell types contribute to the *in vivo* efficacy of mGlu_1_ PAMs also remains an exciting hypothesis. Our phMRI studies suggest that mGlu_1_ PAMs affect multiple brain regions. Actions on mPFC SST-INs may contribute; alternatively, potentiation of mGlu_1_ receptors in other regions may be responsible and could predict mGlu_1_ PAM efficacy across other behavioral modalities. For example, VU6004909 attenuated nucleus accumbens hyperactivity, suggesting that an mGlu_1_ PAM may ameliorate motivational deficits in schizophrenia (Kirkpatrick et al., 2006). Determining the mechanisms of mGlu_1_ activation in other brain regions and potential efficacy in other behaviors relevant to schizophrenia are exciting future directions. The continued development pharmacological and genetic tools to selectively and conditionally study mGlu_1_ will further our understanding of mGlu_1_ biology and its potential as a therapeutic target.

An important limitation of our work is the use of an acute pharmacologically induced deficit model. We sought to specifically model inhibitory deficits (Lewis et al., 2012) and cortical hyperactivity (Manoach et al., 1999) observed clinically in patients with schizophreniaby using acute NMDA receptor antagonism to produce a disinhibition-like state in rodents (Homayoun and Moghaddam, 2007). The etiology of schizophrenia involves a complex interaction of genetic and environmental factors so that preclinical models often cannot recapitulate the entirety of the disease (Jones et al., 2011), and it will be important to test mGlu_1_ PAMs in other schizophrenia-related models. The phMRI experiments were also performed using male rats; it will be critical in the future to determine whether these results are similar in females and in mice as knowledge of potential species or sex differences will be important to guide future development. Additionally, in the current study we investigated mGlu_1_ PAM efficacy after a single, acute dose. An effective symptomatic treatment for schizophrenia will necessitate long-term dosing, and future studies will need to investigate the effects of chronic mGlu_1_ PAM treatment. Finally, although VU6004909 is highly selective for mGlu_1_ (Garcia-Barrantes et al., 2016) and we replicated the efficacy in the Y-maze with a structurally distinct mGlu_1_ PAM, Ro-07-11401, future studies using additional compounds and transgenic approaches will be important to validate on-target efficacy. The profound motor and learning deficits of global mGlu_1_ knockout mice (Aiba et al., 1994a, 1994b) preclude their use for validation of mGlu_1_ PAM on-target activity. Future development of improved genetic approaches to conditionally restrict mGlu_1_ expression will provide opportunities to confirm that mGlu_1_ receptors in specific neurocircuits are required for the behavioral actions of mGlu_1_ modulators.

In this study, we describe the mGlu_1_ receptor as a therapeutic target to preferentially enhance GABAergic transmission and rescue cortical inhibitory deficits. Augmenting receptor function *in vivo* with an mGlu_1_ PAM reversed both cortical hyperactivity and a working memory deficit relevant to cognitive dysfunction in schizophrenia. As mGlu_1_ PAM development continues toward producing a clinical candidate, our study provides preclinical proof of concept that mGlu_1_ PAMs rescue cognitive deficits in addition to their previously observed antipsychotic-like efficacy (Yohn et al., 2020). Based on our mechanistic work, it is possible that mGlu_1_ PAMs will have broad utility in other disorders where I/E balance is perturbed such as epilepsy, autism, and Alzheimer’s disease (Busche and Konnerth, 2016; Fritschy, 2008; Gao and Penzes, 2015). Altogether, these data suggest that mGlu_1_ PAMs have the potential to confer breakthrough efficacy to improve the lives of patients living with schizophrenia.

## STAR★METHODS

### RESOURCE AVAILABILITY

#### Lead contact

Further information and requests for resources and reagents should be directed to and will be fulfilled by the Lead Contact, P. Jeffrey Conn (jeff.conn@vanderbilt.edu).

#### Materials availability

This study did not generate new unique reagents.

#### Data and code availability

- All data reported in this paper will be shared by the lead contact upon request.
- This paper does not report original code.
- Any additional information required to reanalyze the data reported in this paper is available from the lead contact upon request.

### EXPERIMENTAL MODEL AND SUBJECT DETAILS

#### Mice

Adult (> 8 week) transgenic and C57BL/6J mice (Cat No. 000664; RRID:IMSR_JAX:000664) were obtained from Jackson Laboratories (Bar Harbor, ME, USA) and were allowed to acclimate to the housing facility for at least 1 week. SST-Cre::Ai9 and PV-Cre::Ai9 mice were generated by crossing either homozygous SST-Cre mice (Cat No. 028864; RRID:IMSR_JAX:028864) or PV-Cre mice (Cat No. 017320; RRID:IMSR_JAX:017320) with homozygous Ai9 reporter mice (Cat No. 007909; RRID:IMSR_JAX:007909) which carry a Cre-dependent tdTomato allele inserted into the ROSA26 locus. Transgenic mice were previously backcrossed to a congenic C57BL/6J background and mice used for experiments were hemizygous for Cre and heterozygous for the Ai9 reporter allele. Experimental hemizygous SST-Cre mice were generated by crossing homozygous SST-Cre mice with C57BL/6J mice. Littermates of the same sex were randomly assigned to experimental groups. We did not observe any significant effects related to external genitalia; therefore, the data for male and female mice were combined. Mice were cared for in accordance with the National Institutes of Health *Guide for the Care and Use of Laboratory Animals*, were provided with food and water *ad libitum*, and maintained on a 12-hour light/ dark cycle (lights on 6:00 AM). Experiments were performed during the light cycle and all mice were group-housed for the duration of the studies. All mouse experiments were approved by the Institutional Animal Care and Use Committee for Vanderbilt University.

#### Rats

Adult (250-275 g) wild-type Sprague-Dawley rats (RRID:RGD_737903) were obtained from Envigo (Indianapolis, IN, USA) and allowed to acclimate to the housing facility for at least 1 week. Male rats were used for the phMRI studies due to established technical protocols and analysis pipelines and were randomly assigned to experimental groups prior to the procedure. Rats were cared for in accordance with the National Institutes of Health *Guide for the Care and Use of Laboratory Animals*, were provided with food and water *ad libitum*, and maintained on a 12-hour light/dark cycle (lights on 6:00 AM). Experiments were performed during the light cycle and rats were group-housed prior to catheter implantation, then single-housed for the remainder of the experiment. All rat experiments were approved by the Institutional Animal Care and Use Committee for Vanderbilt University.

### METHOD DETAILS

#### Fluorescence *in situ* hybridization

Fluorescence *in situ* hybridization experiments were performed using RNAscope probes and reagents supplied by Advanced Cell Diagnostics (Minneapolis, MN, USA) using the fresh-frozen protocol available online. Probe sets were directed against mouse messenger RNA and included *Grm1* (Cat. No. 449781, channel C1, accession number NM_016976.3, target region 1420-2372), *Slc17a7* (Cat. No. 416631-C3, C3, NM_182993.2, target region 464-1415), *Slc32a1* (Cat. No. 319191-C2, C2, NM_009508.2, target region 894-2037), Sst (Cat. No. 404631-C2, C2, NM_009215.1, target region 18-407), and *Pvalb* (Cat. No. 421931-C3, C3, NM_013645.3, target region 2-885). A set of negative control probes were directed against DapB of *Bacillus subtilis* (Cat. No. 320871).

C57BL/6J mice were anesthetized using 5% isoflurane, decapitated, and brains were rapidly dissected and submerged in ice-cold artificial cerebrospinal fluid (in mM: 126 NaCl, 2.5 KCl, 1.25 Na2PO4, 26 NaHCO3, 10 glucose, 2 CaCl2, 1 MgSO4). Brains were then rapidly frozen in Tissue-Tek O.C.T. Compound using dry ice and stored at −80°C until sectioning. 16 μm, coronal sections containing the mPFC were cut using a Leica Cryostat CM1950 (Leica Biosystems, Buffalo Grove, IL, USA), mounted onto Fisherbrand Superfrost Plus slides (Fisher Scientific), and stored at −80°C until processing. Slides containing mPFC sections were fixed for 15 min in ice-cold 4% paraformaldehyde (PFA), followed by a sequence of dehydration steps by submersion in 50%, 70%, 100%, 100% ethanol at room temperature. Slides were then air-dried and a hydrophobic barrier was drawn around the sections using an ImmEdge PAP Pen (Vector Laboratories, Burlingame, CA, USA). Sections were incubated with Protease IV solution for 30 minutes at room temperature, washed twice in phosphate-buffered saline (PBS) and incubated in a mixture of either*Grm1, Slc17a7, and Slc32a1* or *Grm1, Sst, and Pvalb* RNAScope probes for 2 hr at 40°C in a humidified chamber. Slides were washed in Wash Buffer and then underwent a series of amplification steps at 40°C with AMP 1-FL (30 min), AMP 2-FL (15 min), AMP 3-FL (30 min) and either AMP 4-Alt C-FL (for *Slc17a7/Slc32a1* slides, 15 min) or AMP 4-Alt B-FL (for *Sst/Pvalb* slides, 15 min), washing twice for 5 min in between each incubation. Following a final wash step, sections were incubated with DAPI and then coverslipped using Fluoromount Aqueous Mounting Medium (Millipore-Sigma, St. Louis, MO, USA). Slides were sealed and stored at 4°C until imaging. For each experimental section, a corresponding section from the same mouse underwent the same protocol substituting the 3-plex negative control probe.

Sections were imaged with an inverted Nikon ECLIPSE Ti-E microscope (Nikon Instruments Inc, Melville, NY, USA) using a 20x objective and an Andor Zyla sCMOS camera (Andor USA, Concord, MA, USA). Images were acquired with 405, 488, 561, and 647nm diode lasers and stitched together using NIS-Elements software. Images were analyzed with Fiji software (Schindelin et al., 2012), using the negative control-treated sections to adjust brightness and contrast settings to minimize the visualization of bacterial transcripts and autofluorescence. Regions-of-interest (ROIs) were defined using the C2 or C3 channels and overlaid onto the C1 channel, corresponding to *Grm1* transcript. For each section, the optical density of the C1 channel was calculated within each ROI along with the average optical density of putative single transcripts identified as distinct, round dots with clearly decaying intensity on all sides. Following background correction, the number of “dots per cell” was then calculated by dividing the optical density in a given ROI by the average optical density of a single transcript, or “dot.” A cell was determined to be *Grm1* positive if it contained > 2 dots of *Grm1* transcript within the ROI. Values for “dots per cell” for each hemisphere were averaged together and all statistical comparisons were conducted within animal. For analysis by cortical layer, the distance of the centroid of each ROI was measured from the pia surface and layer boundaries were assigned based on reported values for PL PFC (Anastasiades et al., 2019). All representative images are displayed without the DAPI channel and brightness and contrast were adjusted to improve visualization.

#### Immunofluorescence

Mice were anesthetized with 5% isoflurane, transcardially perfused with ice-cold PBS with 2g/L glucose followed by 4% PFA. Brains were dissected and post-fixed for 24hrs in 4% PFA at 4°C and then washed three times in PBS. Free-floating, 40-60 μm coronal mPFC sections were obtained using a Vibratome (Leica VT1200S, Leica Biosystems, Buffalo Grove, IL, USA) and stored in PBS until processing. Sections were washed with PBS, blocked with 5% normal donkey serum and 0.3% Triton X-100 in PBS for 2 hr at room temperature, and then incubated with primary antibody diluted in blocking buffer overnight at 4°C as follows: goat anti-RFP (Cat. No. 200-101-379, Rockland Immunochemicals, Inc., Limerick, PA) at 1:1000, chicken anti-GFP (Cat. No. ab13970, Abcam, Cambridge, MA, USA) at 1:2000. Slices were washed and then incubated with appropriate secondary antibodies for 2 hr at room temperature as follows at 1:500 dilutions: donkey anti-goat-Cy3 (Cat. No. 705-165-147) and donkey anti-chicken-Alexa488 (Cat. No. 703-545-155, Jackson ImmunoResearch Inc., West Grove, PA, USA). Slices were washed, incubated with DRAQ5 (Cat. No. 4084) for 5 min at room temperature, and then mounted and coverslipped onto Fisherbrand Superfrost Plus slides using Fluoromount Aqueous Mounting Medium. Sections were imaged as detailed for fluorescence *in situ* hybridization experiments.

#### Electrophysiology

Whole-cell patch clamp recordings were performed as previously described (Joffe et al., 2020; Maksymetz et al., 2019). 8-12 week-old mice were anesthetized with 5% isoflurane and the brain was rapidly removed from the skull, blocked, and mounted to the cutting stage of a Vibratome. Coronal sections containing the mPFC were cut at 300 μm and hemisected in ice-cold NMDG-HEPES artificial cerebrospinal fluid (aCSF) containing (in mM): 92 NMDG, 2.5 KCl, 1.25 NaH2PO4, 30 NaHCO3, 20 HEPES, 25 glucose, 2 thiourea, 5 Na-ascorbate, 3 Na-pyruvate, 0.5 CaCl2·2H2O, and 10 MgSO4·7H2O, titrated to pH 7.3-7.4 with HCl and osmolarity adjusted to 300-310mOsm. Slices were then transferred to 32°C for 10-12 min and following recovery, transferred to a holding chamber containing aCSF composed of (in mM): 126 NaCl, 2.5 KCl, 1.25 Na2PO4, 26 NaHCO3, 10 glucose, 2 CaCl2, 1 MgSO4, supplemented with 500μM sodium ascorbate and osmolarity adjusted to 295-300mOsm, at room temperature for a minimum of 1 hr before commencing recording.

For recording, slices were transferred to a submerged recording chamber (Warner Instruments, CT, USA) and perfused with aCSF maintained at 31 ± 1°C using an in-line heater (Warner Instruments, CT, USA) at a rate of 2mL/min. Whole-cell patch clamp recordings were performed in the prelimbic PFC from visually-identified layer V pyramidal neurons or tdTomato-positive SST or PV neurons in response to brief illumination with 565nm light. Recordings of tdTomato-positive neurons from SST::Ai9 mice were discarded if they displayed PV-like fast-spiking and membrane properties (i.e., low input resistance and little to no voltage sag in response to hyperpolarization) due to potential marker expression in PV neurons in this line (Hu et al., 2013). Access resistance, membrane resistance, and holding current were monitored throughout all recordings. Cells where the access resistance changed more than 25% throughout the recording were excluded from analysis.

To record membrane properties, spontaneous excitatory postsynaptic currents (sEPSCs) and current-clamp responses to compound application, pipets pulled to a resistance of 3-5 MΩ were filled with a K-gluconate-based internal solution (in mM: 125 K-gluconate, 4 NaCl, 10 HEPES, 4 MgATP, 0.3 NaGTP, 10 Tris-phosphocreatine). After obtaining a > 1GU seal, fast capacitance was compensated and whole-cell configuration was achieved. Cells were allowed to dialyze for 5 min while voltage clamped at −80 mV. To confirm cellular identity and assess membrane properties and spiking characteristics, the responses to a series of current injections ranging from −150pA to +400pA (25pA increments) were recorded for each cell in current clamp. sEPSCs were then recorded in voltage clamp at −80mV near the reversal potential for GABA_A_ receptor-mediated currents (Joffe et al., 2020). Current-clamp recordings of membrane properties were performed with no current injected and with series resistance compensated for.

To record spontaneous inhibitory postsynaptic currents (sIPSCs) and within-cell ratios of sIPSCs-to-sEPSCs, patch pipets were filled with Cs-based internal solution (in mM: 140 CsMeSO_3_, 5NaCl, 10 HEPES, 0.2 EGTA, 2 MgATP, 0.2 NaGTP, 5 QX-314). sEPSCs and sIPSCs were recorded at −70mV and +10mV, respectively, adjusted for the liquid junction potential. sIPSCs were confirmed to be GABA_A_ mediated as they were abolished by bath application of 50 μM picrotoxin while sEPSCs were eliminated by bath application of 20 μM CNQX and 50 μM D-AP5. For within-cell ratios, baseline sEPSCs were recorded for 2 minutes at −70mV, then voltage was stepped up to +10mV where sIPSCs were recorded at baseline and in response to compound application, then the cell was stepped back to −70mV to obtain a 2-minute sEPSC recording in the presence of test compounds.

For *ex vivo* optogenetic experiments in voltage-clamp, 565nm light (M565L3, Thor Labs, NJ) was delivered through a 40X immersion objective via the epillumination port of an Olympus BX51 inverted microscope with a duration of 100ms at 5Hz to the region around the recording electrode to activate NpHR3.0 for the duration of the compound administration experiments. For current-clamp experiment to verify NpHR-mediated hyperpolarization and inhibition of action potential firing, constant 565nm light was delivered for 2 s or in the same manner used for the voltage-clamp experiments.

All recordings were amplified using an Axoclamp 700B amplifier, digitized with a Digidata 1550B at 20kHz, low-pass filtered at 1.6kHz, and recorded using Clampex 10.7 software (Molecular Devices, San Jose, CA, USA) on a PC running Windows 10. sEPSC and sIPSC characteristics as well as baseline values used to calculate changes in holding current were analyzed using MiniAnalysis (Synaptosoft, Fort Lee, NJ, USA). Membrane properties and action potential characteristics were analyzed using ClampFit 10.7.

For sEPSC, sIPSC, and current-clamp recordings of compound application, baseline and compound values were quantified over a 2-min span directly before addition of compound and at the end of a 5-min compound bath application. For current-clamp recordings of compound application, cells were considered to fire persistent action potentials (APs) if AP frequency was greater than 1 Hz. For membrane properties, analysis was performed similar to other reports (Joffe et al., 2020; McGarry and Carter, 2016). Resting membrane potential (V_m_) was calculated as the average voltage directly preceding a current step for all sweeps. Input resistance was determined by the average steady-state voltage (V_ss_) of the negative current injections. Sag ratio was calculated by the average of (V_sag_ – V_ss_)/(V_sag_ – V_m_) for all negative current steps, where V_sag_ is the peak negative deflection of a negative current step. Adaptation ratio was determined as the average ratio of the last and first interspike intervals across current steps initiating APs where there were more than two spikes, such that larger values indicate greater adaptation.

#### Stereotaxic surgeries and fiber implants

At 5 weeks of age, SST-Cre or SST-Cre::Ai9 mice were anesthetized with 3% isoflurane, positioned in a stereotaxic frame (Kopf Instruments, CA) and maintained on 1%–2% isoflurane for the remainder of the procedure. Briefly, an incision was made and the skin and muscle atop the skull was pulled to the side and cleaned. A bilateral craniotomy was made above the prefrontal cortex. Mice were then bilaterally injected with AAV5-Ef1 α-DIO-eNpHR3.0-eYFP provided as a generous gift from Karl Deisseroth (Addgene viral prep # 26966-AAV5; http://addgene.org/26966; RRID:Addgene_26966) at a volume of 0.6μL of per injection site at a rate of 0.1μL/min using a 28G needle attached to a 10μL Hamilton syringe (Hamilton Co., NV). The needle remained in place for 5 min following injection and was then slowly retracted. For *ex vivo* optogenetic experiments, the mPFC stereotaxic coordinates were (in mm relative to Bregma): AP +1.80, ML ± 0.35, DV −2.00. The scalp was then closed with VetBond (3M, MN), mice were returned to their home cage, and received 10mg/kg carprofen for 48 hours.

For *in vivo* optogenetic experiments, mPFC stereotaxic coordinates were (in mm relative to Bregma, 10° angle): AP + 1.80, ML ± 0.85, DV −2.25. Following bilateral viral injection, two anchoring screws were secured to the caudal part of the skull and two 2mm long, 200 μm core, 0.22 NA fiber optic cannulae (ThorLabs, Newton, NJ, USA) were implanted above the injection sites. Cannulae were secured to the skull with C&B Metabond dental adhesive (Parkell, Edgewood, NY, USA) followed by a layer of dental cement (Integrity Temporary Crown and Bridge Material, Patterson Dental, St. Paul, MN, USA) and the scalp was then closed around the headcap with VetBond. Mice were returned to their home cage and received 10mg/kg carprofen for 48 hours.

#### Pharmacological magnetic resonance imaging

phMRI studies were performed as described previously (Byun et al., 2014; Moehle et al., 2017). Contrast-enhanced cerebral blood volume (CBV) functional MRI was used to obtain *in vivo* measures of drug-induced changes in neural activity. Isoflurane anesthetized Sprague-Dawley rats with preimplanted jugular vein catheters underwent endotracheal intubation (14G plastic catheter), followed by insertion of i.p. and s.c. catheters (22G), and mechanical ventilation (Kent Scientific, Litchfield, CT) delivering isoflurane in a 1:2 O_2_:N_2_O gas mixture. Animals were securely placed in a bite bar (transimaging.com, Raleigh, NC) and pulse rate, respiration rate and pattern, and rectal temperature were continuously monitored (PhysioPro; transimaging.com, Raleigh, NC) and temperature maintained through an air-heating unit (SAM-PC; SA Instruments, Encinitas, CA). End-tidal carbon dioxide was monitored (Invivo Research, Orlando, FL) and maintained. For each scan session, animals were anesthetized under 0.9% isoflurane with neuromuscular blockade (pancuronium bromide, 1 mg/kg). Functional MR images were acquired using a horizontal 9.4T Magnex magnet interfaced with a Varian/Agilent spectrometer and a Doty litz 38-mm transmit-receive radiofrequency coil (Doty). Magnetic field homogeneity was optimized by automatic global shimming followed by local shimming over the rat brain. High-resolution fast spinecho (FSE) anatomical images were collected with the following parameters: repetition time [TR] 2550 ms; effective echo time [TEeff] 40 ms; number of excitations [NEX] 2; 128 × 128 matrix; 35 × 35 mm^2^ field of view [FOV]; 14 1.0 mm thick contiguous slices. Precontrast reference images and post-contrast functional images were acquired (FSE: TR 2600 ms; TEeff 36 ms; NEX 2; 64 × 64 matrix). To determine target engagement and the effects of VU6004909 on the NMDA receptor antagonist-induced regional responses, either vehicle (10% Tween-80 in sterile water) or 60 mg/kg VU6004909 (i.p.) was administered 30 min before scan initiation. To measure changes in CBV, Molday iron oxide nanoparticles (MION, 30 nm; 20 mg/kg, i.v.; BioPAL, Worcester, MA) were injected. After equilibration of MION, post-contrast baseline data were collected for 10 minutes, and then MK-801 was injected (0.3mg/kg, s.c.) and phMRI data were continuously acquired.

phMRI data were first pre-processed using Analysis of Functional NeuroImages (AFNI; https://afni.nimh.nih.gov/). All brain-masked, motion corrected (AFNI 2dreg) images were coregistered to an anatomical template (AFNI 3dreg). Data were processed using in-house MATLAB code (Mathworks); fractional CBV changes were calculated on a voxel-wise basis for each brain slice using the equation ΔCBV(t)/ CBVo = [ln S(t) – ln So]/[ln So – ln Spre], where S(t) is the signal measured at time t, So is the post-contrast baseline signal, and Spre is the pre-contrast baseline signal (Mandeville et al., 1998). Regions of interest (ROIs) that were pre-defined on an anatomical template based on the Paxinos & Watson rat brain atlas (Paxinos and Watson, 2007) were propogated through all slices for all subjects. Mean fractional CBV changes were calculated for each ROI (left / right hemispheres averaged).

#### Animal behavior

Behavioral experiments were performed in adult, C57BL/6J mice with the exception of the *in vivo* optogenetic experiments which were performed in SST-Cre/SST-Cre::Ai9 mice following surgery. Experiments were performed in part through the use of the Murine Neurobehavior Core lab at Vanderbilt University Medical Center.

Spontaneous alternation was performed in a Y-shaped maze with clear, plexiglass walls. Mice were injected with vehicle (10% Tween-80), VU6004909 (60 mg/kg, i.p.), or Ro-07-11401 (30 mg/kg, i.p.) 40 min before injection with either vehicle (0.9% saline) or MK-801 (0.18 mg/kg, i.p) 20 min prior to the start of the behavioral session. Mice were then placed in one arm of the maze, facing away from the center and allowed to freely explore for 8 min. The start arm for each mouse was alternated and the maze was cleaned with 70% ethanol between each mouse. Animal tracking was performed using ANY-maze software with zones for each arm of the maze predetermined. The number and order of entries into each of the three arms were analyzed. A correct spontaneous alternation occurred when the mouse entered a different arm in each of three consecutive arm entries (e.g., ABC or CAB is correct, ABA or CAC is incorrect). Percent alternation was calculated as: (Total correct spontaneous alternations) / (Total arm entries n 2) × 100.

#### *In vivo* optogenetics

*In vivo* optogenetic manipulations of spontaneous alternation performance were performed similar to spontaneous alternation studies in C57BL/6J mice with the following modifications. 6 weeks after viral injection and cannula implantation, mice were habituated to a bifurcated patch cable (BFYL1LF01, Thor Labs). Each fiber optic cannula was wiped clean with 70% isopropyl alcohol and the two ends of the patch cable were connected to the implanted cannulae with ceramic sleeves. Mice were then allowed to explore a novel cage for 5 minutes before being disconnected.

To assess the effects of mPFC SST inhibition on Y-maze performance, mice were randomized to light ON or OFF groups. Both groups were allowed to explore the maze for 8 minutes, split into two, 4 min trials. For the light ON group, the first 4 min were conducted with the light off, then a 560nm LED (Doric Lenses, Quebec, Canada) connected to the other end of the patch cord was turned on for the second 4 min. For the light OFF group, the mice were connected to the patch cord but the light was not turned on. 560nm light (5mW measured at the end of each patch cord cable) was delivered similar to the *ex vivo* slice studies: 100ms duration at 5Hz for 4 min. Mice were placed into an arm of the Y-maze facing away from the center, the start arm was alternated, and the maze was cleaned with 70% ethanol between each mouse.

To assess the effects of mPFC SST inhibition on mGlu_1_ PAM efficacy, mice were injected with vehicle (10% Tween-80) or VU6004909 (60 mg/kg, i.p.) 40 min before injection with either vehicle (0.9% saline) or MK-801 (0.18 mg/kg, i.p) 20 min prior to the start of the behavioral session. All mice were exposed to 560nm light as described above and mice were randomized to have the light ON during the first or second 4-min trial. Mice were initially connected to the patch cable and placed in a novel cage for 30 s. For mice randomized to receive light ON first, the LED was turned on and then mice were then placed into the Y-maze. After 4 min, the LED was turned off. For mice receiving light OFF first, mice were placed into the Y-maze from the novel cage and after 4 min, the LED was turned on.

Videos of the sessions were recorded and total arm entries and order were analyzed by an observer blinded to treatment groups. Zones were marked in the same way as experiments performed in wild-type mice. To control for inaccurate measurements of performance with too few zone entries, mice that failed to perform more than 6 arm entries in either 4-min trial were excluded from analysis. To minimize the number of animals used in this study, mice were returned to their home cage and allowed to recover for 1 week. Mice were then pseudo-randomized to receive a different compound combination for a second test day and we observed no effect of prior exposure to any compound combination on performance in this assay.

Upon completion of the *in vivo* optogenetic experiments, brains were collected and verification of cannula placement and NpHR expression was assessed as described in the immunohistochemistry methods. Mice with improper cannula placement or lack of expression of NpHR in both hemispheres around the cannula tips were excluded from analysis.

#### Compounds

DHPG (S-3,5-dihydroxyphenylglycine) was purchased from Hello-Bio Inc. (Princeton, NJ, USA). (+)-MK-801 maleate, MTEP hydrochloride, and LY367385 were purchased from Tocris Bioscience (Minneapolis, MN, USA). VU6004909 and Ro-07-11401 were synthesized in-house as previously described (Garcia-Barrantes et al., 2016). Stock solutions were prepared in deionized water for DHPG and MTEP, 0.9% saline for MK-801, 1.1 equivalents of NaOH for LY367385, and DMSO (< 0.01% final concentration) for VU6004909. Solutions of VU6004909 from powder or MK-801 from stock solution that were used for behavioral experiments were prepared on the same day as the behavioral task in appropriate vehicles described above.

### QUANTIFICATION AND STATISTICAL ANALYSIS

The number of animals in each experiment is denoted by “N” and the recorded cells by “n.” Data are presented as mean ± standard error (SEM). Statistical analyses and data visualization were performed using GraphPad Prism (La Jolla, CA). A paired or unpaired two-tailed Student’s t test, two-tailed Mann-Whitney test, Fisher’s exact test, one-way ANOVA, or repeated-measures one- or two-way ANOVA with suitable post-tests were used where appropriate. Results of analyses including relevant statistical values and tests are presented in the text or figure legends.

## Supplementary Material

1

## Figures and Tables

**Table:** KEY RESOURCES TABLE

REAGENT or RESOURCE	SOURCE	IDENTIFIER
Antibodies		
Goat anti-RFP	Rockland Immunochemicals	Cat# 200-101-379; RRID: AB_2744552
Chicken anti-GFP	Abcam	Cat# ab13970; RRID: AB_300798
Cy3 donkey anti-goat	Jackson ImmunoResearch	Cat# 705-165-147; RRID: AB_2307351
Alexa Fluor 488 donkey anti-chicken	Jackson ImmunoResearch	Cat# 703-545-155; RRID: AB_2340375
Bacterial and virus strains		
AAV5-Ef1α-DIO-eNpHR3.0-eYFP	Gift from Karl Deisseroth	RRID: Addgene_26966_AAV5
Chemicals, peptides, and recombinant proteins		
DHPG (S-3,5-dihydroxyphenylglycine)	HelloBio	HB0045
(+)-MK-801 maleate	Tocris	0924
MTEP hydrochloride	Tocris	2921
LY367385	Tocris	1237
VU6004909	Garcia-Barrantes et al., 2016	n/a
Ro-07-11401	Vieira et al., 2009	n/a
Critical commercial assays		
RNAscope Fluorescent Multiplex Reagent Kit	Advanced Cell Diagnostics	Cat. No. 320850
Mm-*Grm1*	Advanced Cell Diagnostics	Cat. No. 449781
Mm-*Slc17a7*	Advanced Cell Diagnostics	Cat. No. 416631-C3
Mm-*Slc32a1*	Advanced Cell Diagnostics	Cat. No. 319191-C2
Mm-*Sst*		N/A
Mm-*Pvalb*	Advanced Cell Diagnostics	Cat. No. 421931-C3
3-plex Negative Control (*DapB*)	Advanced Cell Diagnostics	Cat. No. 320871
Experimental models: Organisms/strains		
Mouse: C57BL/6J	The Jackson Laboratories	RRID: IMSR_JAX:000664
Mouse: Sst-IRES-Cre, B6J.Cg-*Sst^tm2.1(cre)Zjh^*/MwarJ	The Jackson Laboratories	RRID: IMSR_JAX:028864
Mouse: PV-Cre, B6.129P2-*Pvalb^tm1(cre)Arbr^*/J	The Jackson Laboratories	RRID: IMSR_JAX:017320
Mouse: Ai9, B6.Cg-Gt(ROSA) *26Sor^tm9(CAG-tdTomato)Hze^*/j	The Jackson Laboratories	RRID: IMSR_JAX:007909
Rat: Hsd:Sprague Dawley SD	Envigo	RRID: RGD_737903
Software and algorithms		
NIS-Elements	Nikon Instruments	https://www.microscope.healthcare.nikon.com/products/software/nis-elements
Fiji	Schindelin et al., 2012	https://imagej.net/software/fiji
pClamp10.7	Molecular Devices	https://mdc.custhelp.com/app/answers/detail/a_id/18779/%7E;/axon%E2%84%A2pclamp%E2%84%A2-10-electrophysiology-data-acquisition-%26-analysis-software-download
MiniAnalysis	Synaptosoft	http://www.synaptosoft.com/MiniAnalysis/
Analysis of Functional NeuroImages (AFNI)	NIH	https://afni.nimh.nih.gov/
MATLAB	Mathworks	https://www.mathworks.com/products/matlab.html
ANY-maze	Stoetling, Co.	https://stoeltingco.com/anymaze.html

## Highlights

- mGlu_1_ augments SST interneuron output to increase prelimbic (PL) cortex inhibition
- An mGlu_1_ positive allosteric modulator (PAM) shifts I-E balance toward inhibition
- An mGlu_1_ PAM reverses MK-801-induced cortical hyperactivity *in vivo*
- mGlu_1_ PAMs reverse working memory deficits via actions on PL SST interneurons
