# Supplementary material for: mGlu1 potentiation enhances prelimbic somatostatin interneuron activity to rescue schizophrenia-like physiological and cognitive deficits
Source: Cell Rep. Author manuscript; Available in PMC 2021 Nov 29. (PMC8628371; doi:10.1016/j.celrep.2021.109950)
Supplement: 1 [file NIHMS1753647-supplement-1.pdf]

Cell Reports, Volume *37*

Supplemental information

mGlu$_1$ potentiation enhances prelimbic somatostatin interneuron activity to rescue schizophrenia-like physiological and cognitive deficits

James Maksymetz, Nellie E. Byun, Deborah J. Luessen, Brianna Li, Robert L. Barry, John C. Gore, Colleen M. Niswender, Craig W. Lindsley, Max E. Joffe, and P. Jeffrey Conn

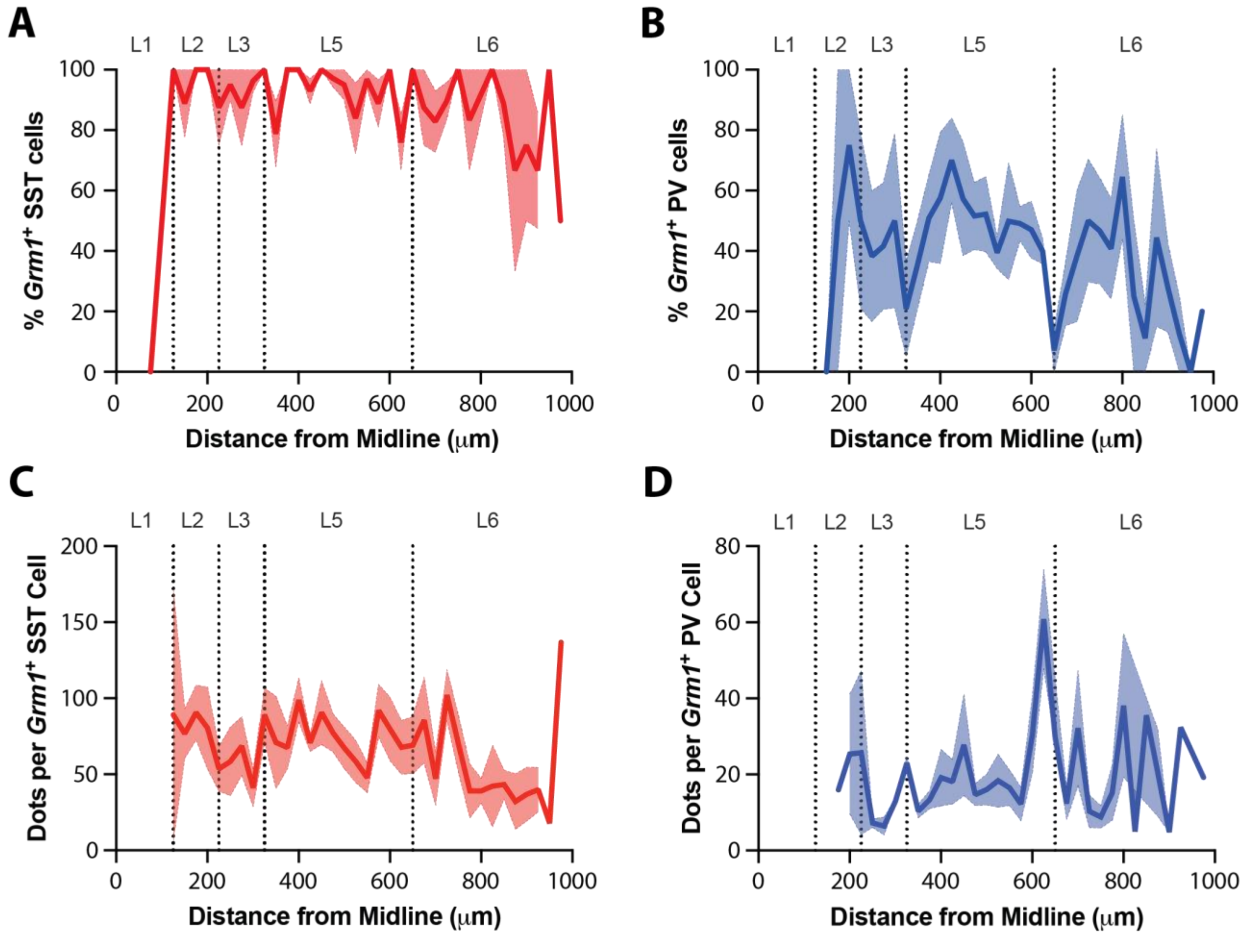

Figure S1. Distribution of *Grm1*-positive SST and PV neurons in the prelimbic PFC by layer, Related to Figure 1.

(A) Distribution of *Grm1*-positive SST cells in the prelimbic cortex as a function of distance from pia surface / midline. (one-way repeated measures ANOVA of values binned by layer, main effect of layer, $F_{(1.075,3.225)} = 0.28$, $p = 0.65$)

(B) Distribution of *Grm1*-positive PV cells in the prelimbic cortex as a function of distance from pia surface / midline. (one-way repeated measures ANOVA of values binned by layer, main effect of layer, $F_{(2.067,6.201)} = 0.26$, $p = 0.78$)

(C) Distribution of *Grm1* expression in SST cells in the prelimbic cortex as a function of distance from pia surface / midline. (one-way repeated measures ANOVA of values binned by layer, main effect of layer, $F_{(1.232,3.697)} = 1.06$, $p = 0.39$)

(D) Distribution of *Grm1* expression in PV cells in the prelimbic cortex as a function of distance from pia surface / midline. (one-way repeated measures ANOVA of values binned by layer, main effect of layer, $F_{(1.243,3.729)} = 2.63$, $p = 0.19$)

N = 4 mice.

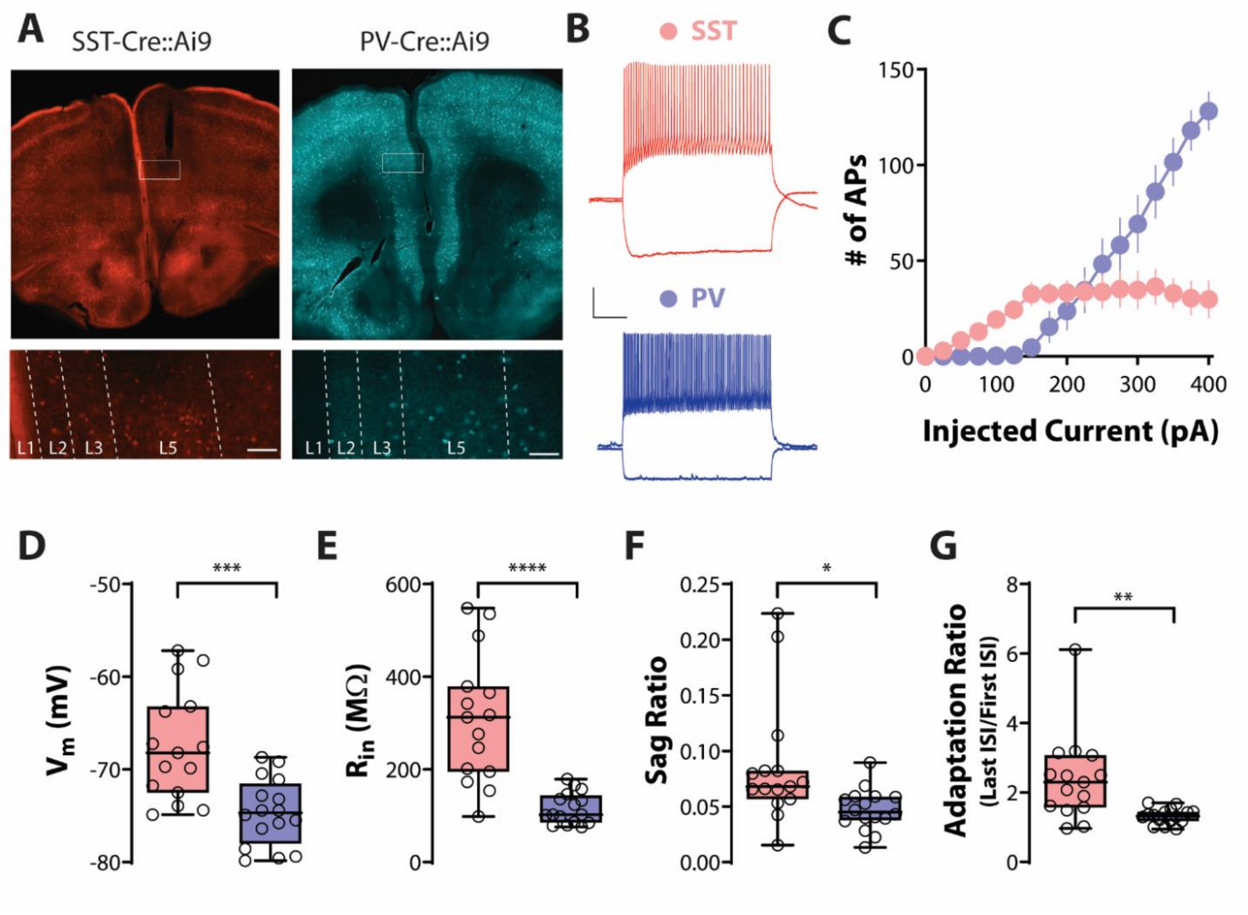

Figure S2. Properties of prelimbic SST and PV interneurons, Related to Figure 2.

(A) Representative images of SST- and PV-IN distribution in coronal slices containing the PFC from SST-Cre::Ai9 and PV-Cre::Ai9 mice, respectively. Bottom, distributions of SST- and PV-INs in the PL cortex, with layer subdivision. Scale bar = 100 µm.

(B) Representative current-clamp traces of the firing properties of SST- and PV-INs.

(C) Input-output curve of action potential (AP) firing in response to positive current injection. APs induced by positive current injection in SST-INs were initiated with less current and plateaued at a lower frequency compared to the fast-spiking output of PV-INs.

(D) SST-INs have a more depolarized resting membrane potential ($p = 0.0003$), **(E)** higher input resistance ($p < 0.0001$), **(F)** larger voltage sag ratio ($p = 0.014$), and **(G)** higher adaptation ratio ($p = 0.002$) compared to PV-INs (two-tailed unpaired Student's t-tests; n/N = 15/7 cells/mice for SST-INs, 16/4 for PV-INs). * $p < 0.05$, ** $p < 0.01$, *** $p < 0.001$, **** $p < 0.0001$.

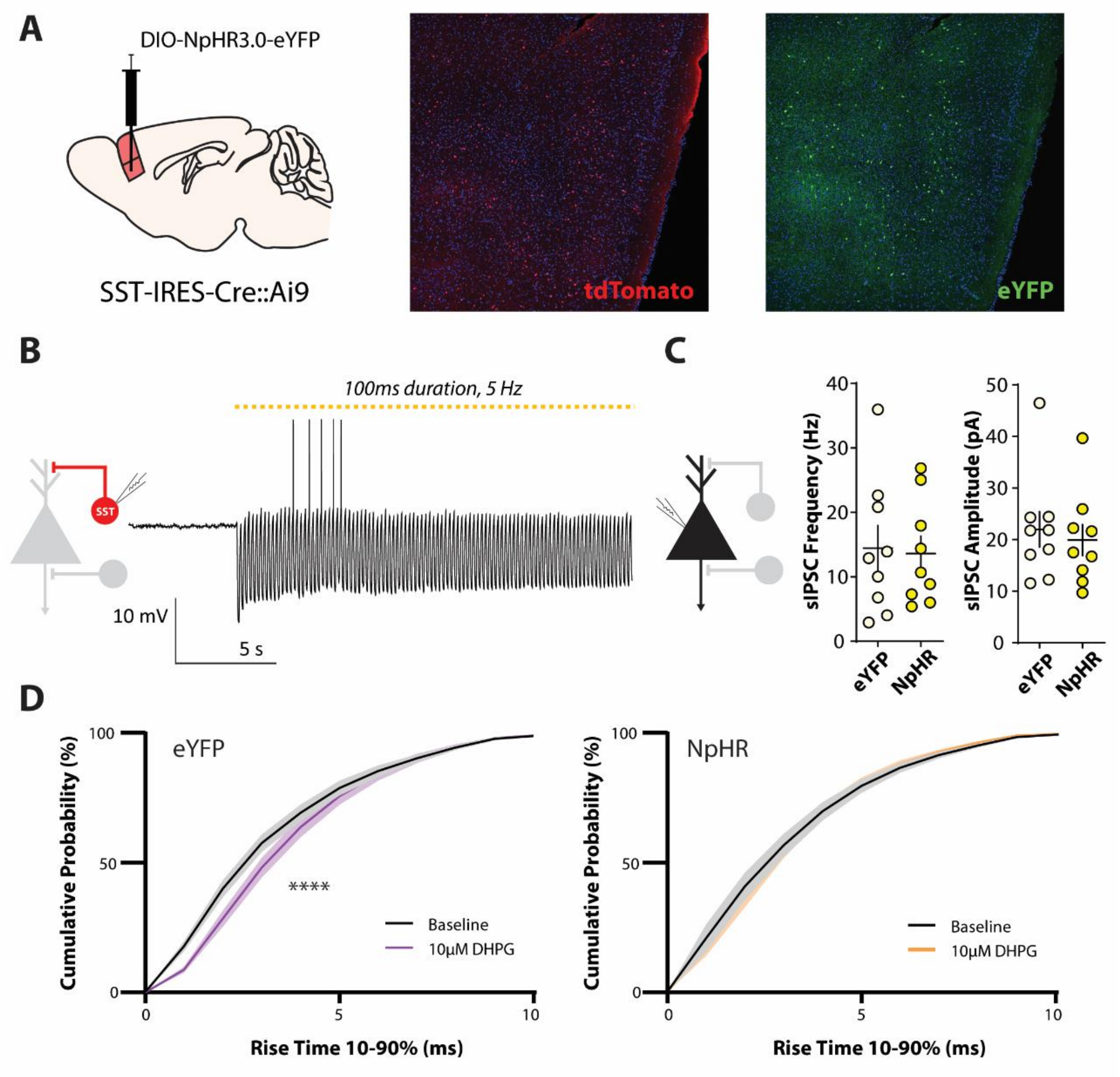

Figure S3. Validation of NpHR3.0-mediated inhibition of PL PFC SST interneurons *ex vivo*, Related to Figure 3.

(A) Schematic depicting the viral injection strategy to selectively express NpHR3.0 in SST-INs in the mPFC. Right, representative images of tdTomato-expression in SST neurons from SST-Cre::Ai9 mice and eYFP-tagged NpHR3.0.

(B) Representative current-clamp recording of a tdTomato-positive/eYFP-positive neuron from an NpHR3.0 infected mouse demonstrating 5Hz 565nm light inhibits SST-INs over an extended period of time.

(C) Baseline sIPSC frequency ($p = 0.853$) and amplitude ($p = 0.660$) recorded in layer V pyramidal neurons are not different between eYFP- and NpHR3.0-infected slices in the presence of 565nm light (n/N = 9/3 cells/mice for eYFP, 9/5 for NpHR3.0).

(D) Cumulative probability plots for eYFP- and NphR3.0-infected slices in response to 10μM DHPG bath application in the presence of 565 nm light (eYFP, two-way repeated measures ANOVA, main effect of drug, $F_{(1,88)} = 17.0$, $p < 0.0001$; NpHR, , two-way repeated measures ANOVA, main effect of drug, $F_{(1,88)} = 0.086$, $p = 0.77$). **** $p < 0.0001$.

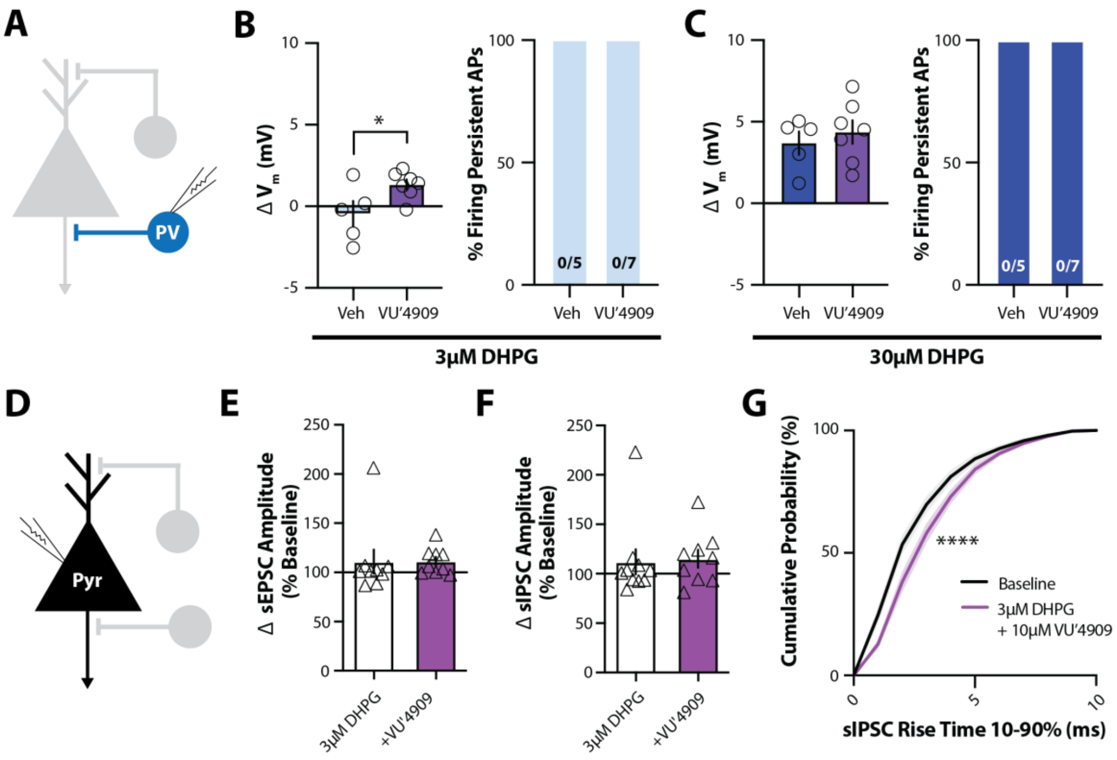

Figure S4. Effects of VU6004909 on prelimbic PV interneurons and layer V pyramidal neurons, Related to Figure 4.

(A) Schematic depicting whole-cell recording of a PV-IN.

(B) *Left,* VU6004909 (10µM) increases PV-IN depolarization by 3µM DHPG (two-tailed unpaired Student's t-test, $p = 0.036$, n/N = 5/4 cells/mice for Veh, 7/5 for VU'4909). *Right*, PV-INs do not fire persistent APs in response to 3µM DHPG alone or with VU'4909. Number of cells responding/total cells denoted. (two-sided Fisher's exact test, $p = 1.00$). * $p < 0.05$.

(C) *Left,* No effect of VU6004909 (10µM) on PV-IN depolarization by 30µM DHPG (two-tailed unpaired Student's t-test, $p = 0.53$, n/N = 5/4 for Veh, 7/5 for VU'4909). *Right*, PV-INs do not fire persistent APs in response to 30µM DHPG alone or with VU6004909 (two-sided Fisher's exact test, $p = 1.00$).

(D) Schematic depicting whole-cell recording of layer V pyramidal neuron.

(E) No difference in sEPSC amplitude in layer V pyramidal neurons in response to bath application of 3µM DHPG with and without 10µM VU6004909 (two-tailed unpaired Student's t-test, $p = 0.98$, n/N = 9/4 for 3µM DHPG, 9/4 for +VU'4909).

(F) No difference in sIPSC amplitude in layer V pyramidal neurons in response to bath application of 3µM DHPG with and without 10µM VU6004909 (two-tailed unpaired Student's t-test, $p = 0.85$, n/N = 10/8 for 3µM DHPG, 9/4 for +VU'4909). **** $p < 0.0001$.

(G) Cumulative probability plot of sIPSC rise time during baseline and after bath application of 3µM DHPG + 10µM VU6004909 (two-way repeated measures ANOVA, main effect of drug, $F_{(1,88)} = 118.3$, $p < 0.0001$).

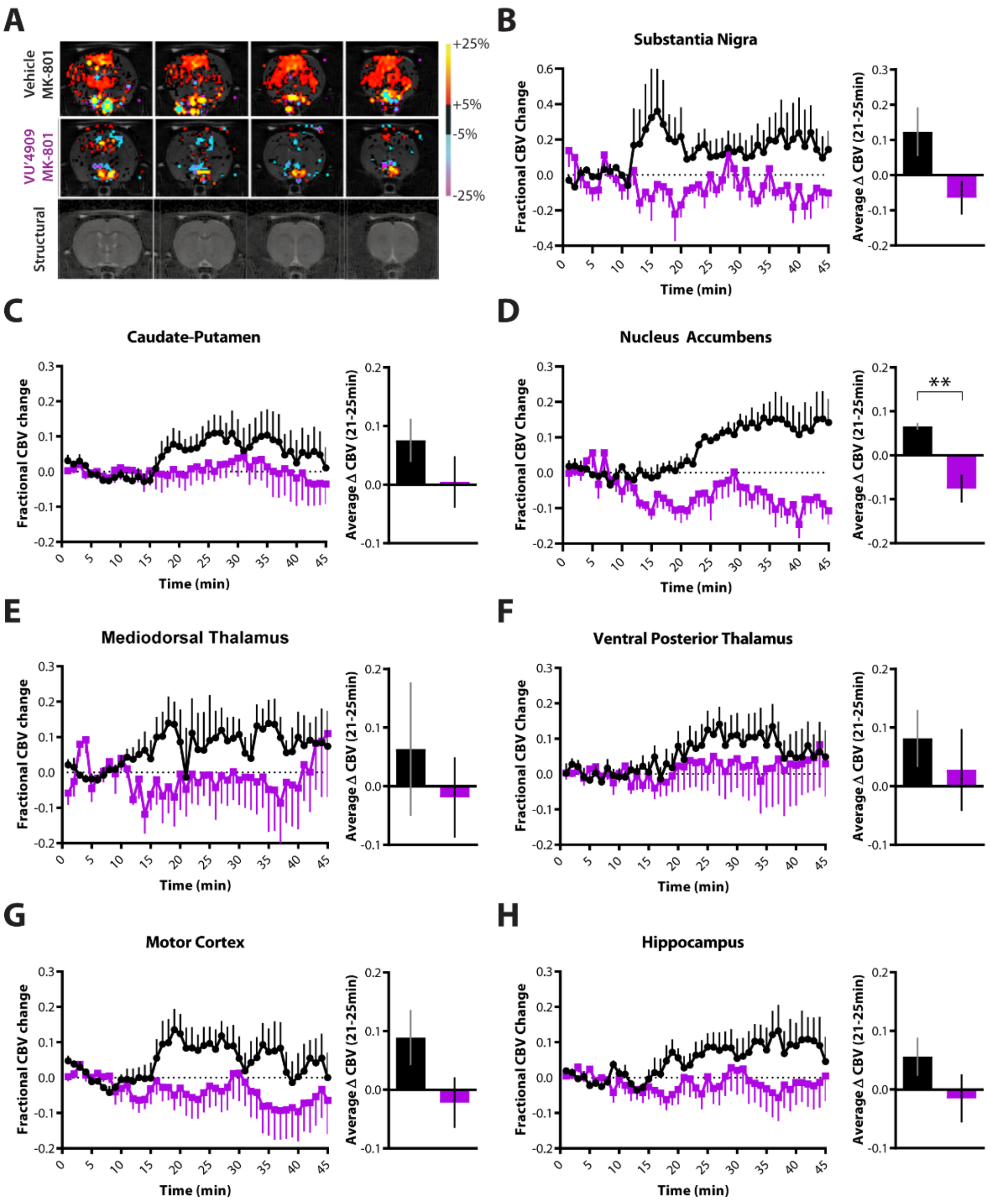

Figure S5. phMRI results of MK-801 and VU6004909 effects in non-cortical brain regions, Related to Figure 5.

(A) Representative CBV and structural T2-weighted MRI template images.

(B-H) Time courses and bar graphs of CBV changes after MK-801 injection (arrow) in rats pretreated with vehicle or VU6004909. 60 mg/kg VU6004909 reverses MK-801-induced hyperactivity in the nucleus accumbens ($p = 0.0022$, $N = 6$ rats per group). ** $p < 0.01$.

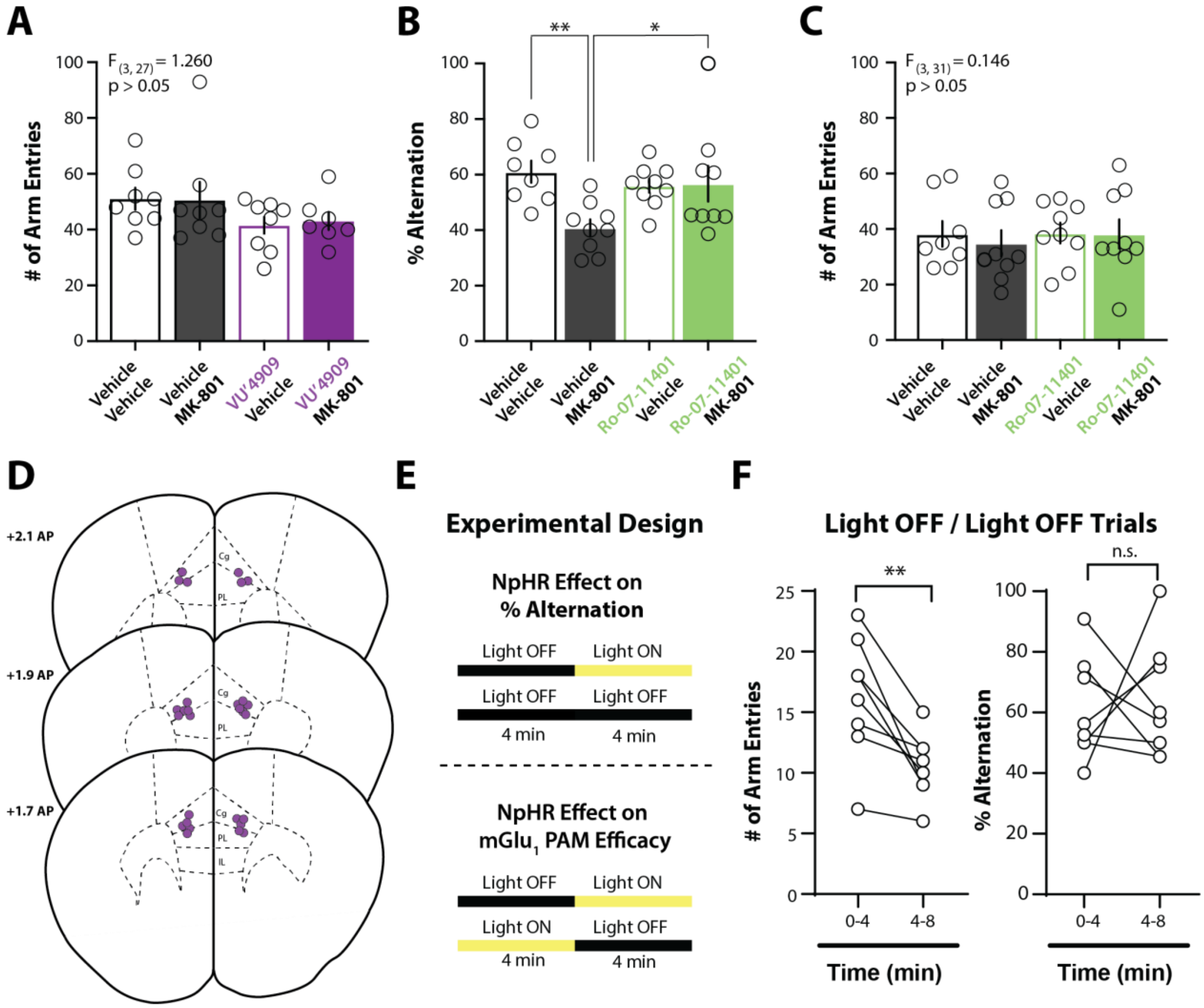

Figure S6. A structurally-distinct mGlu$_1$ PAM rescues MK-801-induced deficits in spontaneous alternation and control data for *in vivo* optogenetic experiments, Related to Figure 6.

(A) Neither 0.18 mg/kg MK-801 (*i.p.*, 0.9% saline), 60 mg/kg VU6004909 (*i.p.*, 10% Tween-80), nor the combination affects number of arm entries in the Y-maze (one-way ANOVA, $F_{(3,27)}$ = 1.260, p = 0.31; N = 8 mice for Vehicle/Vehicle, 8 for Vehicle/MK-801, 8 for VU'4909/Vehicle, 7 for VU'4909/MK-801).

(B) Average % spontaneous alternation in mice pretreated with vehicle or 30 mg/kg Ro-07-11401 (i.p., 10% Tween-80) prior to administration of vehicle or 0.18 mg/kg MK-801 (i.p., 0.9% saline) 20 minutes before behavioral test. MK-801 impairs spontaneous alternation and this deficit is reversed by Ro-07-11401 (one-way ANOVA main effect, $F_{(3,31)}$ = 4.31, p = 0.012; post-hoc Bonferroni's test: Vehicle/Vehicle vs. Vehicle/MK-801, p = 0.0072; Vehicle/MK-801 vs. Ro-07-11401/MK-801, p = 0.035; Vehicle/Vehicle vs. Ro-07-11401/Vehicle, p = 1.00; N = 8 for Vehicle/Vehicle, 9 for Vehicle/MK-801, 9 for Ro-07-11401/Vehicle, 9 for Ro-07-11401/MK-801).

(C) Neither 0.18 mg/kg MK-801 (*i.p.*, 0.9% saline), 30 mg/kg Ro-07-11401 (*i.p.*, 10% Tween-80), nor the combination affects number of arm entries in the Y-maze (one-way ANOVA, $F_{(3,31)}$ = 0.146, p = 0.93).

(D) Fiber optic cannula tip placement for the *in vivo* SST-IN NphR3.0 experiment, as described in Fig 6C-F. Cg = cingulate cortex, PL = prelimbic cortex, IL = infralimbic cortex, AP = anterior-posterior coordinates in mm, relative to Bregma.

(E) Schematic depicting the experimental design for the two *in vivo* optogenetic experiments. To assess if inhibiting PFC SST-INs affects Y-maze performance, mice either performed the entire 8 min session with the light OFF or 4 min with the light OFF followed by 4 min of light ON. To test if PFC SST-INs are required for $mGlu_1$ PAM efficacy, all mice performed 4 min light ON and 4 min light OFF but the order was randomized.

(F) Control data from Light OFF/OFF trials showing that arm entries decrease over the 8 minute session (two-tailed paired t-test, $p = 0.0041$) but performance does not ($p = 0.79$, $N = 8$ mice).
